# Expansion and Assessment of a Web-Based 24-Hour Dietary Recall Tool, Foodbook24, for Use Among Diverse Populations Living in Ireland: Comparative Analysis

**DOI:** 10.2196/52380

**Published:** 2025-02-07

**Authors:** Grace Bennett, Shuhua Yang, Laura A Bardon, Claire M Timon, Eileen R Gibney

**Affiliations:** 1 UCD Institute of Food and Health University College Dublin Dublin Ireland; 2 Quadram Institute Bioscience Norwich Research Park Norwich United Kingdom; 3 School of Population Health Royal College of Surgeons Dublin Ireland

**Keywords:** dietary assessment, ethnic diets, dietary records, web-based tools, diverse intake

## Abstract

**Background:**

Currently, the methods used to collect dietary intake data in Ireland are inflexible to the needs of certain populations, who are poorly represented in nutrition and health data as a result. As the Irish population is becoming increasingly diverse, there is an urgent need to understand the habitual food intake and diet quality of multiple population subgroups, including different nationalities and ethnic minorities, in Ireland. Foodbook24 is an existing web-based 24-hour dietary recall tool, which has previously been validated for use within the general Irish adult population. Because of its design, Foodbook24 can facilitate the improved inclusion of dietary intake assessment in Ireland.

**Objective:**

We aimed to examine the suitability of expanding the Foodbook24 tool, improving the reliability and accuracy of dietary intake data collected among prominent nationalities in Ireland.

**Methods:**

This study consisted of three distinct parts: (1) expansion of Foodbook24, (2) testing its usability (ie, acceptability study), and (3) examining the accuracy (ie, comparison study) of the updated Foodbook24 tool. To expand Foodbook24, national survey data from Brazil and Poland were reviewed and commonly consumed food items were added to the food list. All foods were translated into Polish and Portuguese. The acceptability study used a qualitative approach whereby participants provided a visual record of their habitual diet. The comparison study consisted of one 24-hour dietary recall using Foodbook24 and one interviewer-led recall completed on the same day, repeated again 2 weeks later. Comparison study data were analyzed using Spearman rank correlations, Mann-Whitney *U* tests, and κ coefficients.

**Results:**

The expansion of the Foodbook24 food list resulted in 546 additional foods. The acceptability study reported that 86.5% (302/349) of foods listed by participants were available in the updated food list. From the comparison study, strong and positive correlations across 8 food groups (44% of a total of 18 food groups) and 15 nutrients (58% of a total of 26 nutrients) were identified (*r*=0.70-0.99). Only intakes of *potatoes and potato dishes* and *nuts, herbs, and seeds* significantly differed across methods of assessment, where correlations across these food groups were low (*r=*0.56 and *r=*0.47, respectively). The incidence of food omissions varied across samples, with Brazilian participants omitting a higher percentage of foods in self-administered recalls than other samples (6/25, 24% among the Brazilian vs 5/38, 13% among the Irish cohort).

**Conclusions:**

The updated food list is representative of most foods consumed by Brazilian, Irish, and Polish adults in Ireland. Dietary intake data reported in Foodbook24 are not largely different from food groups and nutrient intakes reported via traditional methods. This study has demonstrated that Foodbook24 may be appropriate for use in future research investigating the dietary intakes of Brazilian, Irish, and Polish groups in Ireland.

## Introduction

### Background

Following a call from the European Food Safety Authority seeking “initiatives to further develop web-based tools in the area of dietary surveys,” the development of technology-based dietary assessment tools has greatly increased [1]. Traditional paper-based methods of dietary assessment are inflexible, costly, and labor intensive for both participants and researchers [2,3]. Many recently developed web-based tools offer improved accuracy and provide researchers with additional flexibility to capture data from those living abroad, who have reduced mobility or limited access to transport, or who are not proficient in the same language as the researcher [4-6]. Although digital advances have improved how dietary intake is assessed, systematic errors such as recall bias, lack of diversity within food lists, lack of flexibility for different languages, and inaccurate food portion size estimates remain [7-9]. Similar to many high-income countries across Europe and worldwide, the demographic landscape of Ireland is changing rapidly [10,11]. Not only is the Irish population aging, but also it is becoming more culturally diverse, with the number of different ethnic groups living in Ireland growing by approximately 20% annually [10,12]. As a result, the country has been enriched with an increased array of languages, religions, and cultural and dietary practices. Despite this, it is widely accepted that ethnic minority groups remain underrepresented in social and nutritional science research [13,14]. Ensuring diversity in ongoing data collection is important to understand current dietary patterns of different population subgroups and identifying if targeted interventions are required to limit diet-associated health risks.

### Diversity in Nutrition Data

Food-based dietary guidelines primarily rely on data from national food consumption surveys, but methods of dietary assessment used in these surveys are often not adapted to include diverse languages and foods consumed by different nationalities. In essence, national food consumption surveys tend to underrepresent specific population subgroups [15]. Some national food consumption surveys look to cater to diverse population groups by offering alternative languages for specific samples: in the United States, the National Health and Nutrition Examination Survey food frequency questionnaire is also translated into Spanish for Hispanic participants, and in Switzerland, the menuCH 24-hour dietary recall is available in German, French, or Italian; however there is no such dietary assessment tool available for the diverse population in Ireland [16,17]. In Ireland, adult national food consumption surveys are conducted every 10 years and while data on ethnicity are collected, very poor diversity is achieved; therefore, little is known about the dietary intakes of the ethnic minority groups in Ireland and how they compare to each other and to the healthy eating guidelines.

Foodbook24, a web-based 24-hour dietary recall tool, was originally developed to accurately assess dietary intakes of the Irish adult population living in Ireland [18,19]. As with many web-based dietary assessment tools, participants select foods consumed from a prepopulated food list; nutrient compositions are based on the United Kingdom “Composition of Food Integrated Database” (CoFID), and food images are used to estimate portion sizes [19]. Foodbook24 has been previously validated against 4-day food diaries, interviewer-led 24-hour dietary recalls, and biomarkers of dietary intake (ie, using plasma and urine samples) [19,20]. Foodbook24 was designed for the general Irish population: English speakers who follow a typical “Western” diet, and the original food list was based on foods reported by Irish adults as part of the National Adult and Nutrition Survey; therefore, its use in population subgroups such as specific nationalities living in Ireland who consume a diverse range of food items is limited.

### Objectives

To further enhance the capabilities of the existing tool and ensure it is appropriate for use among different nationalities living in Ireland, Foodbook24 was further developed to include more languages, including Brazilian Portuguese and Polish, and food items commonly consumed by Polish and Brazilian adults. This study aimed to examine the suitability of expanding the Foodbook24 tool for use among diverse nationalities in Ireland and assess the accuracy of the dietary intake data collected via Foodbook24 by comparing it to traditional assessment methods, ensuring dietary intake of the ethnic minority groups can be investigated using appropriate assessment tools.

## Methods

### Selection of Diverse Population Groups

The samples selected within this study were chosen to ensure representation of the current population of Ireland. Irish adults were included as a reference sample, as the tool was previously shown to be comparable within this sample [1]. Renewed comparison among this group would ensure the food list was still reflective of Irish dietary habits today. Polish and Brazilian samples were chosen on the basis of their prevalence in Ireland, differing native languages, and unique dietary traditions [12]. According to the latest available census from 2016, the largest population group in Ireland apart from Irish and Irish Traveler was the Polish population, who equated to 2.6% (122,515/4,761,865) of the total Irish population [12]. Other than British and Eastern European residents, who follow similar dietary patterns to Irish and Polish groups, Brazilians were the next most common non-Irish nationality living in Ireland (approximately 14,000 nationals) [12]. In addition to a large number of people from these countries living in Ireland, these samples are distinctly and culturally different from Ireland and from each other in terms of their native tongue and habitual dietary intake.

**Figure 1:**
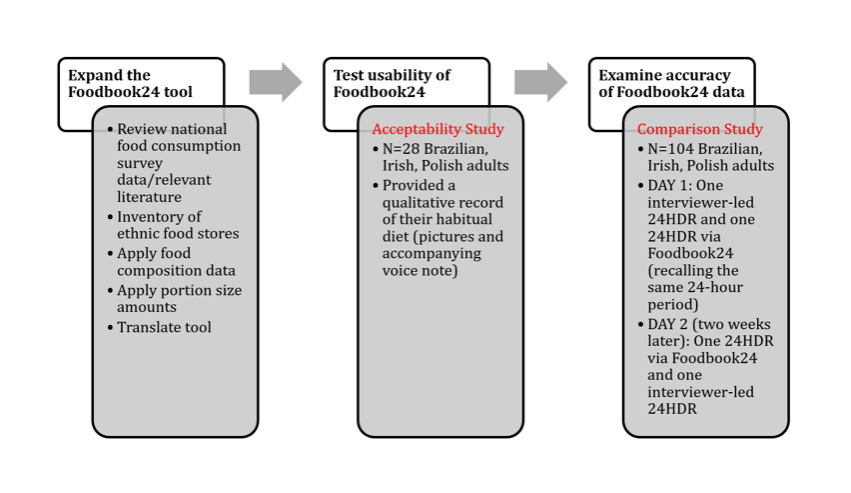
Summary of the 3 phases of Foodbook24 expansion. 24HDR: 24-hour dietary recall.

This study consisted of three distinct parts: (1) expansion of Foodbook24, (2) testing the usability of the Foodbook24 tool (ie, acceptability study), and (3) examining the accuracy of the updated Foodbook24 tool (ie, comparison study). Each of these sections are described in detail in the subsequent sections, individually. Figure 1 provides a summary of each part of the Foodbook24 expansion and testing as well as key activities within each phase.

### Expansion of Foodbook24

Expansion of Foodbook24 was conducted in a multistage process to ensure it could accurately cater for specific population groups (ie, diverse nationalities, vegans, and vegetarians). The first stage of expanding Foodbook24 was to identify and add food items and beverages frequently consumed by Brazilian and Polish adults according to national food consumption surveys and relevant literature from each country [21-29]. The food list in Foodbook24 was also updated to ensure that it fully reflects the current food consumption habits of Irish adults. The original food list in Foodbook24 was based on foods reported in National Adult and Nutrition Survey and had not been updated for the Irish adult population since 2017 [18,19]. On the basis of recent literature and findings from the Foodbook24 acceptability study, there has been a rise in the consumption of certain food products, as well as vegetarianism and veganism, in Ireland. These new-to-market foods were also added to the Foodbook24 food list to account for their increased consumption in Ireland. Next, nutrient composition data were applied to the newly added foods. The Foodbook24 food list primarily consists of individual food items, rather than composite meals, allowing participants to account for cultural-specific recipes and meal ingredients. As the foods listed in Foodbook24 are available on the Irish market, CoFID nutrient compositions were applied to foods where possible [30]. National nutrient composition databases from Brazil and Poland were used for foods consumed by these populations where nutritional information did not exist in CoFID (ie, foods which are likely more culturally specific and less widely available in Ireland) [31,32]. For a limited number of non-Polish or Brazilian food items added to the Foodbook24 food list, predominantly those considered new to the food market (eg, many milk alternatives such as oat or almond), no appropriate composition could be found within CoFID; therefore, average food label information from 5 branded sources were used. Portion size estimation was then applied to new foods; portion size estimates were sought from national food consumption surveys, and if unavailable, portion size estimates of the closest alternative food or drink item from the Ministry of Agriculture, Fisheries and Food’s “Food Portion Sizes” book were used [33]. From national food consumption surveys, the mean reported intake was defined as the medium portion size. One SD was then added to this to determine the medium-large portion size, 2 SDs were added for the large portion size, and 3 SDs were added for the extra-large portion. To calculate smaller portion sizes, increasing SDs were subtracted from the mean reported intake (ie, mean–1 SD, mean–2 SD, and mean–3 SD for the medium-small, small, and extra small portion sizes, respectively). For some foods that were consumed less frequently, the SD was larger than the mean reported intake; therefore, the smaller and larger portion sizes were calculated as the mean +25% to –25%. Existing portion size images were used where possible for the newly added food and drink items. Where there were no appropriate pre-existing images, new food portion size images were taken following a standardized procedure. Finally, all aspects of the Foodbook24 tool were translated and tested by native Brazilian Portuguese and Polish speakers.

### Acceptability Study Design

To test the completeness of the expanded food list, an acceptability study was conducted. The main aim of the acceptability was to understand habitual diets of diverse nationalities living in Ireland and assess the ease at which these diets would be captured using Foodbook24. Participants (n=28) were asked to complete a collage of their habitual diet using the web-based application Pinterest (Pinterest, Inc) and record an accompanying voice note providing further details of the food items and beverages reported within the Pinterest board such as cooking method, condiments added, and whether they were homemade or ready-made food items. This qualitative method of describing habitual diet, commonly termed as *photovoice*, has been previously used in the form of focus groups to identify food choice and practices of underrepresented communities [34,35]. As this study was conducted during the COVID-19 lockdown, it was not possible to conduct focus groups; therefore, the approach using Pinterest was preferred. For the analysis of the acceptability data, the food content of each Pinterest board was extracted, along with the accompanying voice notes, which were transcribed verbatim for clarity and to support the content. Two researchers (GB and SY) extracted data collected from the acceptability study and formed a list of the final food items reported by participants. All authors reviewed this list and a consensus was then reached as to what foods to add to Foodbook24 on the basis of (1) the frequency each food item was reported and (2) the similarity of each food to the ones existing in Foodbook24 (ie, foods deemed similar were not added as separate food items but were “tagged” to existing foods).

### Comparison Study Design

The same protocol that was originally used to assess the use of Foodbook24 among the Irish adult population was applied to this study (Figure S1 in Multimedia Appendix 1) [20]. At baseline, participants (n=104) completed one self-administered 24-hour dietary recall using Foodbook24 and one interviewer-led 24-hour dietary recall with a trained researcher on the same day. The order in which recalls were completed was randomized, with 75% (78/104) of the participants completing the Foodbook24 recall first. Two weeks later, the process was repeated in the opposite order to the first dietary recall. Interviewer-led recalls were conducted by 2 researchers with a strong background in nutrition or dietetics using the Zoom platform (Zoom Communications, Inc) because of COVID-19 pandemic restrictions (GB and SY). Researchers followed a predefined protocol to ensure consistency across interviewer-led recalls, which was developed in accordance with the automated multiple pass method by the US Department of Agriculture [36]. Through previous research activities, researchers conducting the interviewer-led recalls had a strong understanding of dietary cultures of each sample group. All interviews were conducted in English. During the interviewer-led recalls, Foodbook24 portion size images within a food atlas were shown to participants using the “share screen” function on Zoom to estimate portion size using the same method as the self-administered method. If a food item mentioned by participants during the interviewer-led recall was not available in the food list, portion size images of a similar food item were used instead. For example, the portion size image for “white rolls” was shown to participants when the food item *pan de queso* (ie, cheese bread) was mentioned. Participant intake data collected through interviewer-led recalls were recorded on paper. The data were subsequently entered by the same researcher who facilitated the interviewer-led recall into Foodbook24 so that the 2 methods could be compared; by re-entering interviewer-led recalls into Foodbook24, no differences were found in portion size estimates or food composition data. This was important to ensure that the differences in reported intakes could be explained by different assessment approaches rather than other aspects of the 24-hour dietary recalls; the previous Foodbook24 comparison study also used this method [20]. Participants also completed demographic and tool evaluation questionnaires as part of this study.

### Participant Recruitment

**Figure 2:**
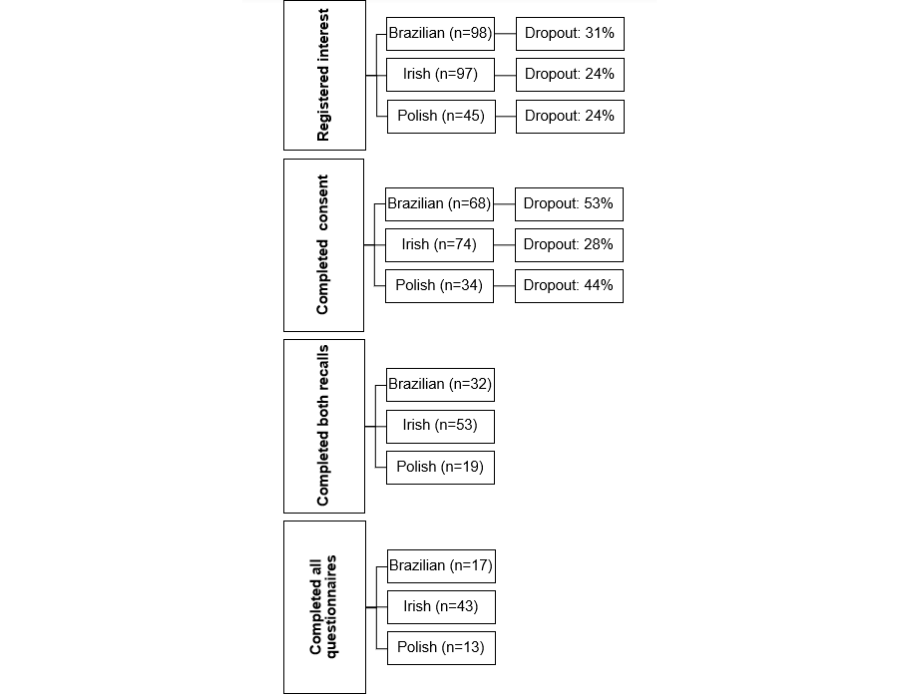
Participant interest and retention at each stage of the comparison study.

Recruitment for both acceptability and comparison studies took place between January 2021 and May 2022. During recruitment, authors hoped to achieve an equal distribution of Brazilian, Irish, and Polish participants across representative age and sex categories. In total, 132 participants were recruited, including 37 (28%) Brazilian, 74 (56.1%) Irish, and 21 (15.9%) Polish. Social media, in-person, and web-based avenues were used to recruit target groups. To attract attention from within the community at University College Dublin (UCD), study posters were displayed around the campus and recruitment events hosted. Other relevant in-person events across Dublin were also attended, such as a Brazilian craft fair and an exhibition for older adults in Dublin. Study information in the form of flyers was distributed outside food shops and restaurants owned by Polish and Brazilian populations. Information posters were also hung on noticeboards of churches, where services were held in Polish, and community centers. Study posters and flyers were printed with an active QR code so that participants could sign up and begin the study directly. Study information was also shared on the web on the UCD Institute of Food and Health and Irish Volunteer websites. Study-specific Facebook (Meta Platforms, Inc) and Instagram (Meta Platforms, Inc) accounts were created to attract attention among target groups, and advertisements were posted on Facebook. Participants were deemed eligible once they identified as a Brazilian, Irish, or Polish adult (aged ≥18 years), lived in Ireland, spoke basic English (ie, able to communicate with researchers during interviewer-led recalls through English), and did not hold a formal degree in nutrition or dietetics. An overview of participant recruitment and retention numbers for the comparison study is available in Figure 2.

### Statistical Analysis

Descriptive analysis was performed on qualitative acceptability study data to understand the food type and frequency of reporting among target groups of interest. Foods recorded by participants in the acceptability study were compared to foods listed in Foodbook24. Mean daily energy, macro- and micronutrient, and food group intakes from the comparison study were compared across the 2 methods (ie, self-administered and interviewer-led recalls). The relationship between both methods was compared using Spearman rank correlations and percentage differences [(self-administered intake − interviewer-led intake) / (self-administered) × 100]. Data were not normally distributed, (ie, according to the Kolmogorov-Smirnov test; *P*<.001); thus, nonparametric tests were used for analysis. Mann-Whitney *U* tests were performed to identify significant differences between methods (ie, *P*≤.05). Statistical significance was assessed using unadjusted and Bonferroni-corrected *P* values [35]. Bland Altman analysis was conducted for key nutrients and food groups to estimate the interval of agreement (95% CI), where the self-administered nutrient value was subtracted from the interviewer-led nutrient value. Relative agreement of the 2 recall methods was assessed using cross-classification analysis. Agreement across methods was also assessed by examining the frequency of food matches, omissions, and intrusions rates for the total population and individual samples [20,36,37]. Exact matches were items that were identical in both recalls, and total matches included exact matches as well as those that were a similar match (eg, low-fat vs full-fat milk). Omissions were defined as items recorded in the interviewer-led recall but not the self-administered recall, and intrusions were those reported in the self-administered recall but not the interviewer-led recall [20]. Misreporters are removed from nutritional data to provide mean intakes of a sample who provided a likely accurate report of what they ate during the 24-hour period. To identify intake underreporters, estimated energy requirements were calculated by multiplying the basal metabolic rate (ie, based on the Henry equation) by 1.1 [38]. Participants whose mean intakes were below their estimated energy requirements for both interviewer-led and self-administered 24-hour dietary recalls were deemed to be underreporters. Only data for adequate reporters are presented here; however, results for the total population including underreporters and individual cohorts are available in Tables S1-S15 in Multimedia Appendix 1. Overreporters were also considered in this research by applying energy intake cutoffs, but none were identified in this sample. All data analyses were performed using SPSS Statistics (version 27; IBM Corp), and statistical significance was set at *P*<.05.

### Ethical Considerations

Ethics approval for both the acceptability and comparison studies was provided by the Office of Research Ethics in UCD (LS-20-48-Gibney). All participant information leaflets and consent forms were provided to participants in English and their native language of Brazilian or Polish. Within the participant information leaflets, participants were informed what data would be collected and why, how these data would be handled and used (ie, pseudonymized immediately once collected), and how they could withdraw themselves and their data from the study if desired. Participants could only participate in this research once the consent form was complete. All participant data was fully de-identified before analysis where each participant was given an unique identifier code. There were no financial incentives for participants to take part in this research. Participants were offered personalized dietary feedback after their completion of the study.

## Results

### Foodbook24 Expansion

During this phase of Foodbook24 development, a total of 546 new foods were added to the Foodbook24 food list; of these, 420 (76.9%) were ethnic foods and 126 (23.1%) were additional Irish foods. The 24-hour dietary recalls in Foodbook24 can now be accurately completed in English, Polish, and Portuguese where participants can select their language of preference. All data outputs available to researchers are presented in English.

### Acceptability Study

In total, 28 participants completed the acceptability study; of these, 21 (75%) were Irish, 5 (18%) were Brazilian, and 2 (7%) were Polish. The mean age of the total sample was 37.71 (SD 16.48) years, with participants from Polish and Brazilian samples aged between 27 and 41 years. In total, 349 items were recorded by participants in the acceptability study, including 65 (18.6%) from *breads, potatoes, and grains*; 40 (11.5%) from *fats, sugars, and desserts*; 17 (4.9%) from *dairy*; 82 (23.5%) from *protein foods*; 78 (22.3%) from *fruit and vegetables*; and 67 (19.2%) from the *other* group. Of the 349 items mentioned, 302 (86.5%) were available in the Foodbook24 food list or a similar alternative to the reported item was available (eg, *garlic mayonnaise* did not exist in the food list but could be substituted for *mayonnaise, regular fat*). In this scenario, names of items not explicitly included in the food list but compositionally very similar to existing foods were added as *search tags* to these original items within Foodbook24: search tags are linked to food items in Foodbook24 to support participants to find foods quickly; search tags include brand names or common colloquial terms for food items. Frequently reported food and drink items, which were not explicitly named in Foodbook24, were predominately from the *soups, sauces, and miscellaneous* and *cheese* groups (14/44, 32% and 3/10, 30% of reported foods from these groups, respectively, were not included in the food list). From the *soups, sauces, and miscellaneous* group, condiments such as coconut oil and peri peri sauce were added as *search tags* as suitable alternatives were available in the Foodbook24 food list. These foods, which were similar to existing foods in terms of type and composition, were not added to the updated Foodbook24 food list to keep the Foodbook24 tool concise and not to overwhelm or confuse participants when selecting food items. Edam and Gouda were popular cheeses reported by Brazilian and Polish adults; as these items were not specifically mentioned in the food list, they were added as new foods.

### Comparison Study

In total, 104 participants were recruited to take part in the comparison study, including 53 (51%) Irish, 32 (31%) Brazilian, and 19 (18%) Polish participants. The mean age of the total sample was 38.83 (SD 14.19) years, 72% (75/104) of which were female participants (Table 1). Findings from the total population are presented in Tables 2-4. Findings for each individual sample (Brazilian, Irish, and Polish) are available in Tables S2-S4 in Multimedia Appendix 1. Comparing demographic characteristics across sample groups, Brazilian and Polish groups were predominately female (28/32, 88%) and 79% (15/19) of Brazilian and Polish adults were aged between 30 and 45 years (18/32, 56% and 12/19, 63% respectively). The Irish sample was more evenly distributed in terms of sex (32/53, 60% female) and age (7/53, 13% aged 30-45 years, ranging from 18 to 72 years). Full demographic data were obtained for 84 participants (30/85, 36% underreporters; 54/85, 64% adequate reporters; and no overreporters were identified), where 74% (63/85) have completed third-level education (minimum of primary bachelor’s degree). Of the total sample, 30 (29%) were considered underreporters and so were excluded from results reported in Tables 2-5. Comparing demographic characteristics between adequate and underreporters, underreporters were more likely to be male (underreporters 13/30, 43% vs adequate reporters 13/54, 24%), older (mean age of under reporters43.07, SD 15.21 years vs mean age of adequate reporters=36.54, SD 13.86 years), and have a third-level education (underreporters 24/30, 80% vs adequate reports 39/54, 72%; Table S1 in Multimedia Appendix 1). Underreporters were more likely to have overweight status (mean BMI 26.39 kg/m^2^) compared to adequate reporters whose mean weight status was in the normal category (mean BMI 23.41 kg/m^2^; Table S1 in Multimedia Appendix 1).

Total sample mean nutrient intakes from the self-administered Foodbook24 method were compared to mean nutrient intakes from the interviewer-led method, excluding underreporters (Table 2). Minimal differences in key nutrient intakes were observed between both methods. Vitamin A was the only nutrient where a statistically significant difference was observed, whereby mean vitamin A intake via the interviewer-led method was significantly higher than the self-administered recall (mean 1050.05, SD 589.57 µg/day vs mean 907.47, SD 702.54 µg/day; *P*=.03; *r*=0.61). Moderate to high associations, using Spearman rank correlation analysis, for all nutrients were found between both recall methods with correlations ranging from 0.51 to 0.77 (Table 2).

**Table 1:** Participant demographics of the total sample from the comparison study.

Participant characteristics		Total sample	Brazilian	Irish	Polish	*P* value	
Population, n (%)^a^		104 (100)	32 (21.8)	53 (36.1)	19 (12.3)	—^b^	
Age (y), mean (SD)^a^		38.83 (14.2)	33.19 (5.8)	41.89 (18.1)	39.89 (8.1)	.02	
**Sex, n (%)^a^**							.09
	Male	29 (27.9)	4 (12.5)	21 (40.0)	4 (21.0)		
	Female	75 (72.0)	28 (87.5)	32 (60.0)	15 (79.0)		
BMI (kg/m^2^)^c^		23.51	22.40	23.67	24.88	.53	
**Education, n (%)^c^**							.21
	Level ≥8^d^	63 (74.0)	16 (73.0)	36 (75)	11 (79.0)		
	Other^e^	22 (26.0)	6 (27.0)	12 (25)	3 (21.0)		
**Physical activity, n (%)^c^**							.06
	≤2 times, weekly	35 (42.0)	11 (50.0)	20 (43.5)	4 (29.0)		
	≥3 times, weekly	48 (58.0)	11 (50.0)	26 (56.5)	10 (71.0)		

^a^Percentage of participants within the total sample, not those who completed the full demographic questionnaire.

^b^Not applicable.

^c^BMI, education, and physical activity data were provided by 84 participants who completed the full demographic questionnaire.

^d^Primary or postgraduate university degree.

^e^Leaving certificate degree (high school equivalent), diploma, or apprenticeship.

**Table 2:** Mean energy and nutrient intakes of adequate reporters recorded via interviewer-led and self-administered methods (comparison study).

Nutrients	Interviewer-led intakes (n=74)^a^, mean (SD)	Self-administered intakes (n=74)^b^, mean (SD)	*P* value^c^	Difference (%)	Correlations (*r*^d^)
Energy (kcal/day)	2000.95 (526.66)	2077.61 (724.51)	.71	3.69	0.63
Protein (g/day)	87.56 (36.83)	83.86 (30.61)	.72	–4.40	0.52
Carbohydrate (g/day)	235.05 (76.64)	245.54 (91.23)	.54	4.27	0.64
Sugars (g/day)	96.20 (46.27)	100.30 (64.11)	.86	4.08	0.77
Starch (g/day)	130.53 (45.21)	130.99 (51.64)	.99	0.36	0.58
Dietary fiber (g/day)	18.19 (6.92)	17.84 (6.80)	.83	–1.93	0.62
Total fat (g/day)	82.44 (23.82)	84.17 (27.46)	.81	2.05	0.64
Saturated fat (g/day)	32.00 (12.66)	31.72 (14.63)	.70	−0.86	0.77
Monounsaturated fat (g/day)	29.51 (9.41)	29.39 (11.18)	.89	−0.38	0.68
Polyunsaturated fat (g/day)	6.40 (2.68)	6.88 (3.56)	.77	6.93	0.51
Protein (% energy)	17.74 (6.13)	16.59 (4.67)	.45	−6.96	0.63
Carbohydrate (% energy)	43.80 (7.01)	44.28 (6.87)	.60	1.10	0.68
Total fat (% energy)	37.28 (6.11)	37.14 (7.06)	.90	−0.38	0.75
Vitamin D (µg/day)	7.48 (8.37)	7.95 (9.41)	.72	5.89	0.70
Vitamin E (mg/day)	15.39 (12.25)	14.35 (10.52)	.58	−7.24	0.70
Vitamin B6 (mg/day)	4.91 (10.66)	4.29 (8.53)	.97	−14.43	0.71
Vitamin B12 (µg/day)	19.51 (115.67)	14.69 (58.66)	.62	−32.80	0.59
Folate (mg/day)	350.28 (224.29)	347.15 (240.54)	.58	−0.90	0.79
Vitamin C (mg/day)	208.51 (234.55)	152.23 (192.50)	.13	−36.97	0.63
Calcium (mg/day)	992.25 (382.92)	923.89 (399.07)	.20	−7.40	0.65
Magnesium (mg/day)	332.42 (110.58)	353.96 (164.43)	.76	6.08	0.68
Phosphorus (mg/day)	1353.13 (357.09)	1370.17 (541.70)	.72	1.24	0.68
Iron (mg/day)	19.07 (22.03)	18.40 (19.90)	.83	−3.65	0.63
Iodine (mg/day)	161.23 (94.58)	145.24 (93.79)	.20	−11.01	0.69
Vitamin A (µg/day)	1050.05 (589.57)	907.37 (702.54)	.03^e^	−15.73	0.61
Carotene (µg/day)	3934.75 (3227.69)	3288.09 (3483.47)	.06	−19.67	0.56

^a^Interviewer-led 24-hour dietary recall.

^b^Self-administered 24-hour dietary recall (via Foodbook24).

^c^*P* values represent significant differences between methods (Mann-Whitney *U* tests); significance at <.05 level.

^d^All *P* values for Spearman rank correlations were <.001.

^e^Only significant for unadjusted *P* values following Bonferroni correction.

**Table 3:** Mean food group intakes of adequate reporters recorded via interviewer-led and self-administered methods (comparison study).

Food group (g/day)	Interviewer-led intakes (n=74)^a^, mean (SD)	Self-administered intakes (n=74)^b^, mean (SD)	*P* value^c^	Difference (%)	Correlations (*r*^d^)
Beverages^e^	997.14 (894.54)	926.92 (1357.55)	.07	−7.58	0.67
Biscuits, cakes, and buns	46.89 (35.62)	51.63 (48.26)	.96	9.19	0.75
Breads, rolls, and scones	74.94 (43.41)	66.06 (43.78)	.21	−13.45	0.63
Breakfast cereals	103.22 (84.83)	104.09 (85.84)	.99	0.83	0.96
Butter, spreads, and oils	11.16 (6.96)	10.85 (7.69)	.55	−2.87	0.51
Cheese	27.20 (21.76)	20.99 (13.58)	.19	−29.58	0.53
Creams, ice creams, and desserts	65.64 (58.84)	51.45 (47.69)	<.001^f^	−27.59	0.71
Egg and egg dishes	107.64 (85.38)	105.25 (100.42)	<.001^f^	−2.27	0.76
Fish and fish dishes	64.67 (55.92)	73.38 (64.87)	.06	11.87	0.69
Fruit and fruit juices	271.09 (251.96)	266.29 (218.23)	<.001^f^	−1.80	0.86
Grains, rice, pasta, and savories	161.28 (105.43)	174.78 (100.52)	.02^g^	7.72	0.58
Meat and meat products	155.41 (94.98)	163.81 (97.66)	.38	5.13	0.68
Milk and yoghurts	140.26 (108.36)	145.00 (139.50)	.06	3.27	0.66
Nuts, herbs, and seeds	9.57 (7.08)	14.09 (10.74)	<.001^f^	32.07	0.47
Potatoes and potato dishes	98.62 (51.71)	112.30 (66.97)	<.001^f^	12.18	0.56
Soups, sauces, and miscellaneous	96.88 (110.01)	97.31 (110.58)	0.01^g^	0.44	0.74
Sugars, confectionary, and preserves	38.35 (35.15)	39.97 (38.78)	0.02^h^	4.05	0.86
Vegetables and vegetable dishes	200.35 (126.47)	182.61 (147.38)	<.001^h^	−9.71	0.79

^a^Interviewer-led 24-hour dietary recall.

^b^Self-administered 24-hour dietary recall (via Foodbook24).

^c^*P* values represent differences between methods (Mann-Whitney *U* tests).

^d^All *P* values for Spearman rank correlations were <.001.

^e^Water removed from *beverages* food category.

^f^Only significant for unadjusted *P* values following Bonferroni correction.

^g^Significant for adjusted and unadjusted *P* values following Bonferroni correction.

^h^Only significant for unadjusted *P* values following Bonferroni correction.

**Table 4:** Cross-classification of mean food group intakes of adequate reporters recorded via self-administered and interviewer-led methods (comparison study).

Food group	κ coefficient	Exact^a^ (%)	Adjacent^b^ (%)	Disagreement^c^ (%)	Extreme disagreement^d^ (%)
Beverages^e^	0.50	62.16	28.38	6.76	2.70
Biscuits, cakes, and buns	0.48	64.86	27.03	5.41	2.70
Breads, rolls, and scones	0.33	52.70	36.49	6.76	4.05
Breakfast cereals	0.87	91.89	8.11	0	0
Butter, spreads, and oils	0.46	63.51	22.97	9.46	4.05
Cheese	0.45	67.57	20.27	6.76	5.41
Creams, ice creams, and desserts	0.49	79.73	13.51	2.70	4.05
Egg and egg dishes	0.73	90.54	4.05	4.05	1.35
Fish and fish dishes	0.60	85.14	13.51	0	1.35
Fruit and fruit juices	0.71	78.38	18.92	1.35	1.35
Grains, rice, pasta, and savories	0.46	59.46	28.38	9.46	2.70
Meat and meat products	0.34	51.35	36.49	9.46	2.70
Milk and yoghurts	0.33	56.76	25.68	12.16	5.41
Nuts, herbs, and seeds	0.46	68.92	24.32	2.70	4.05
Potatoes and potato dishes	0.56	72.97	16.22	6.76	4.05
Soups, sauces, and miscellaneous	0.38	55.41	36.49	8.11	0
Sugars, confectionary, preserves	0.47	62.16	36.49	1.35	0
Vegetables and vegetable dishes	0.45	58.11	35.14	6.76	0

^a^Percentage of cases cross-classified into the same quartile.

^b^Percentage of cases cross-classified into an adjacent quartile (+1 or −1).

^c^Percentage of cases cross-classified 2 quartiles apart.

^d^Percentage of cases cross-classified 3 quartiles apart.

^e^Water removed from the *beverage* food group.

**Table 5:** Percentage of matches, omissions, and intrusions observed between interviewer-led and self-administered recalls across samples (comparison study).

	Total match^a^, n (%)	Exact match^b^, n (%)	Omissions^c^, n (%)	Instrusions^d^, n (%)
Brazilian (n=25)	17 (69)	13 (52)	6 (23)	2 (8)
Irish (n=38)	31 (81)	25 (67)	4 (12)	3 (7)
Polish (n=11)	8 (74)	6 (56)	2 (18)	1 (8)
Total sample (n=74)	55 (74)	43 (58)	13 (18)	6 (8)

^a^Total matches include items that were identical as well as similar, for example, whole meal bread and granary bread were recorded as similar matches.

^b^Exact matches represent items that were identical only.

^c^Omissions account for items reported in the interviewer-led recall but not the self-administered recall.

^d^Instrusions include items reported in the self-administered recall but not the interviewer-led recall.

**Figure 3:**
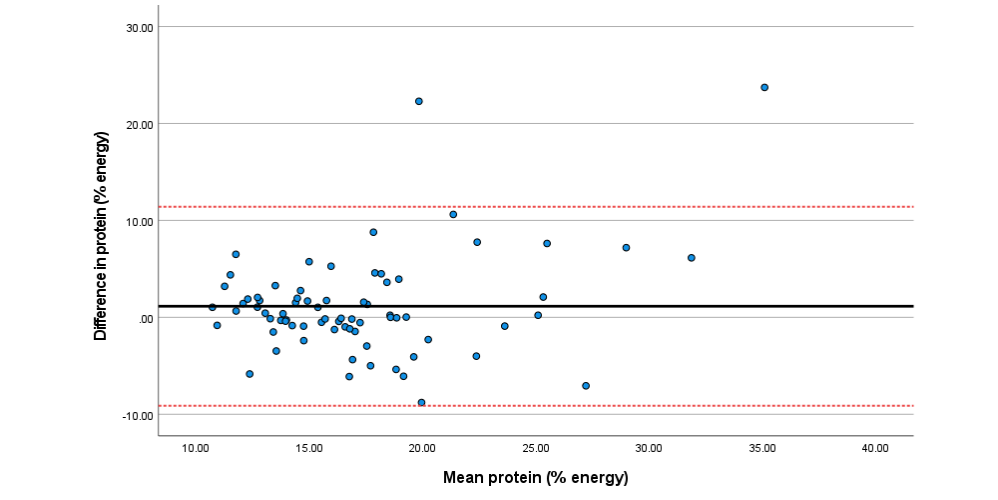
Difference in protein intake (percentage total energy) reported in the self-administered and interviewer-led recall.

**Figure 4:**
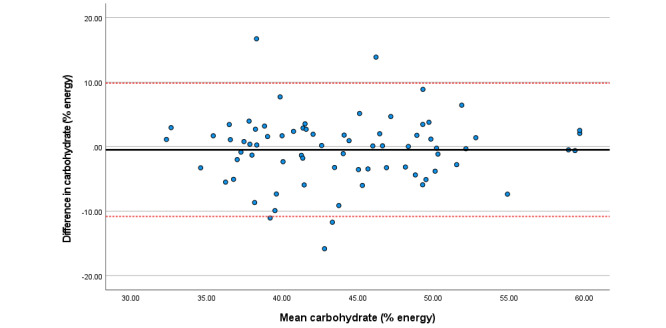
Difference in carbohydrate intake (percentage total energy) reported in the self-administered and interviewer-led recall.

**Figure 5:**
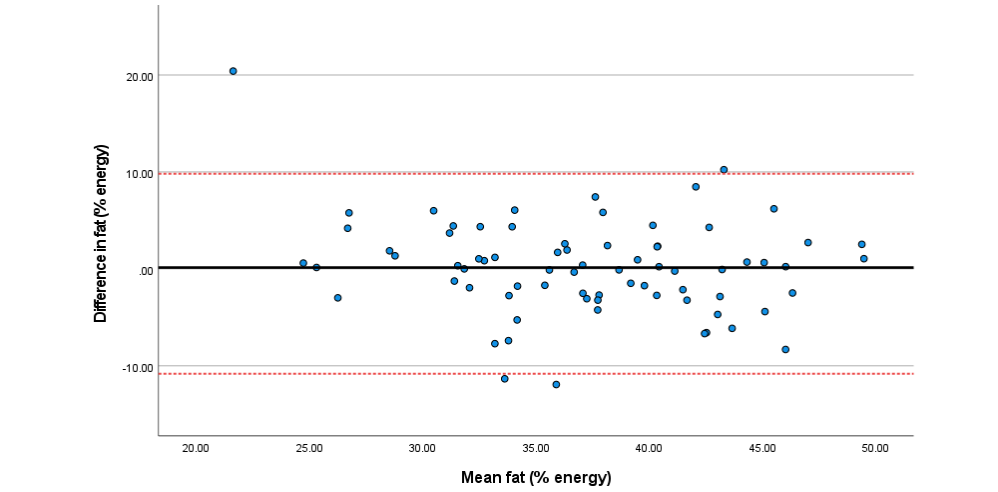
Difference in fat intake (percentage total energy) reported in the self-administered and interviewer-led recall.

**Figure 6:**
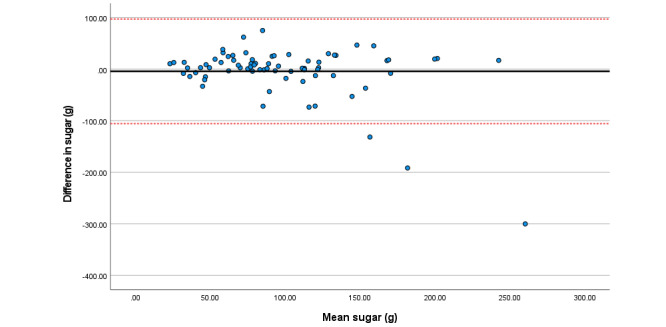
Difference in sugar intake (g/day) reported in the self-administered and interviewer-led recall.

**Figure 7:**
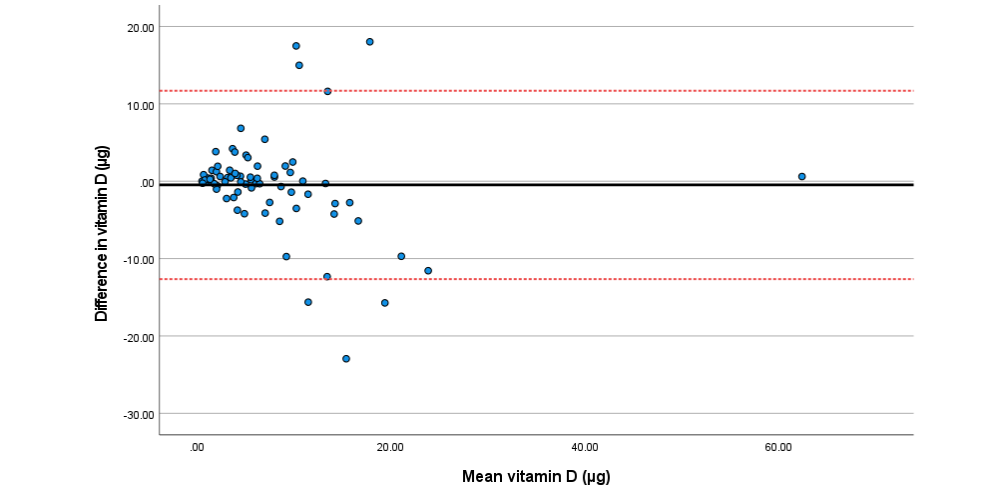
Difference in vitamin D intake (µg/day) reported in the self-administered and interviewer-led recall.

**Figure 8:**
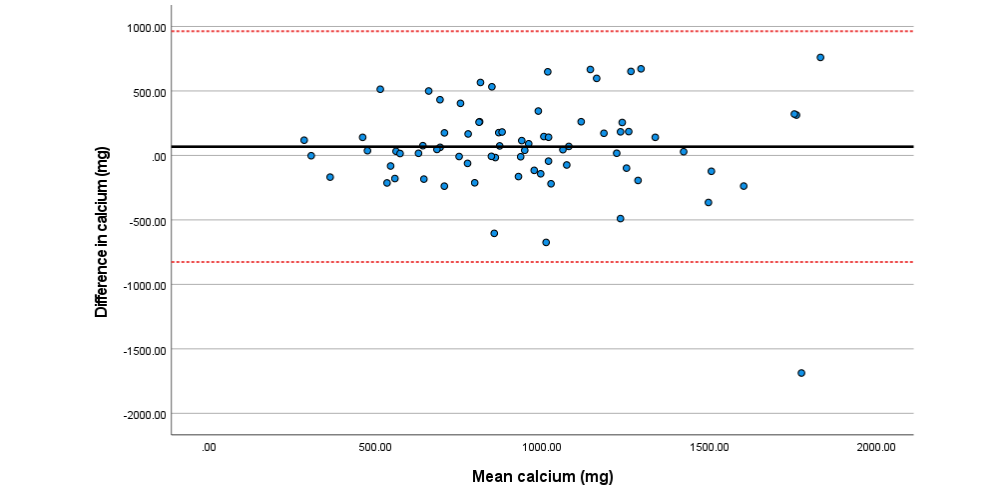
Difference in calcium intake (mg/day) reported in the self-administered and interviewer-led recall.

Within the total sample, associations among total sugars, fat (as a percentage of energy), saturated fat, and folate were particularly strong. Bland Altman plots display agreement across key nutrients (Figures 3-8). Good agreement was found for protein (percentage of total energy), sugar, and calcium (Figures 3, 6, and 8, respectively) with <5% of participants falling outside the limits of agreement. Approximately 5% of participants fell outside the limits of agreement for carbohydrate (% energy) and total fat (% energy); however, correlations for these nutrients were moderate (*r*=0.6) and high (*r*=0.90), respectively (Figures 4 and 5; Table 2).

Analysis of each individual sample is available in Tables S2-S4 in Multimedia Appendix 1; no significant differences were observed for any nutrient among all samples. Correlations across nutrients within the Irish sample were high, with moderate to high correlations observed for nutrient intake with the Brazilian sample. Poor correlations (*r*<0.30) were reported for vitamin B12, iodine, vitamin A, and carotene intakes within the Polish sample (Table S3 in Multimedia Appendix 1). Good agreement across individual intake was found across all individual samples for protein, fat, and carbohydrate (as percentage energy); sugar; vitamin D; and calcium (Figures S1-S3 in Multimedia Appendix 1).

Tables 3 and 4 provide an overview of mean population and individual food group intakes across the 2 recall methods. In total, intake of 6 food groups differed significantly across the interviewer-led and self-administered recalls, once adjusted. Of these 6 groups, potatoes and potato dishes and nuts, herbs, and seeds were poorly correlated with high percentage differences observed across recall methods for the nuts, herbs, and seeds group (*r*=0.56, *P*<.001, percentage difference=12.18 and *r*=0.47, *P*<.001, percentage difference=32.07, respectively; Table 3). Similar trends were identified within individual sample groups, with Brazilian, Irish, and Polish participants overestimating intake of the nuts, herbs, and seeds group by up to 40% and low correlations between methods were observed across each sample for this food group (*r*=0.39-49; Tables S9-S11 in Multimedia Appendix 1).

Determining cross-classification of food group intakes, all food groups had >80% of respondents in the same or adjacent quartiles, with the *breakfast cereals*; *fish and fish products*; *fruit and fruit juices*; and *sugars, confectionary, and preserves* groups having >95% of records in the same or adjacent quartiles (Table 4). κ coefficients indicate moderate agreement for the *beverages*, *fish and fish products*, and *potatoes and potato dishes* groups; good agreement for the *fruit and fruit juices* and *egg and egg dishes* groups; and excellent agreement for the *breakfast cereals* group where all participants were within the same or adjacent quartile. Less than 15% of participant recalls were ≥2 quartiles apart for all food groups except for the *milk and yoghurts* group (Table 4).

The number of matches, omissions, and intrusions of food items are outlined by sample in Table 5. All food and drink items except water were included in this analysis. Rates of matches, omissions, and intrusions were similar across all 3 samples, with Irish participants least likely to omit food items compared to Brazilian and Polish groups (12% vs 23% and 18%, respectively). Food items most frequently omitted by participants across all groups were sauces and condiments from the *soups, sauces, and miscellaneous* group. Intrusions were most likely to be food items from the *sugars, confectionary, and preserves* group such as biscuits and chocolate.

## Discussion

This study has shown that Foodbook24 is a useful method for capturing food group and nutrient intakes in a multinational population living in Ireland. Intakes recorded using Foodbook24 are comparable to those recorded through interviewer-led 24-hour dietary recalls.

### Principal Findings

Findings from the qualitative aspects of the Foodbook24 acceptability study demonstrated that most foods consumed by Irish, Brazilian, and Polish adults living in Ireland are available in the Foodbook24 tool and highlighted additional food items that are often consumed by target groups in Ireland. Although this study offered unique insights into the habitual food intake of diverse population subgroups in Ireland, a small sample size of Brazilian and Polish adults was achieved, meaning that the updated Foodbook24 food list may not be wholly representative of the diets of Brazilian and Polish populations in Ireland.

Results from the comparison study indicated similarity in reported food group and nutrient intakes between Foodbook24 and interviewer-led recalls. Comparison studies of similar tools have reported some differences in energy and key micronutrients, including in the original comparison of Foodbook24; however, these were considered small and often insignificant, much like the results presented by Timon et al [20], Foster et al [39], Albar et al [40], and Labonté et al [41]. In line with this study, previous comparison studies of the Foodbook24, Automated Self Administered 24-hour Dietary Assessment Tool (ASA24), and INTAKE24 tools also examined match, omission, and intrusion rates of foods recorded across 2 methods and, similar to the findings reported here, found comparably high match rates (ie, approximately 80%) [20,39,42]. Although statistically significant differences were reported across mean food group intakes among the total sample, the actual differences were minimal and correlations across methods were relatively high within the context of each food group. An exception to this was the *nuts, herbs, and seeds* food group where population and individual intakes indicated low to moderate agreement; overall low consumption of this food group may have exaggerated small differences in intake across methods. Although previous work reported minimal differences in intakes recorded via Foodbook24 and interviewer-led methods for the *nuts, seeds, herbs, and spices* group, this food group has been identified as difficult to quantify using other 24-hour dietary assessment tools, such as INTAKE24 [9,20]. As this food group is typically consumed in small amounts (serving size=30 g), estimating its portion size can be difficult, and the percentage difference may be exaggerated (eg, a 5 g difference between two methods). Altering how the portion size of this food group is recorded, such as recording the number of nuts or teaspoons of seeds, herbs, and spices consumed rather than using weighed portion size food images, may help improve accuracy of reporting for this food group.

Findings in mean food group and nutrient intakes within the Irish, Brazilian, and Polish sample groups are similar to those reported for the total sample. Irish-specific findings align with those previously identified during the initial Foodbook24 comparison study, where high correlations across self-administered and interviewer-led methods were observed [20]. Despite slightly lower correlations among Polish and Brazilian groups, no significant differences were found across intakes and similar intakes across methods indicate suitability of Foodbook24 for assessing dietary intake of Polish and Brazilian adults in Ireland.

### Impact of Work

As populations in the developed world continue to diversify, it is essential that tools are developed and validated to be flexible for use by key minority groups of the population. Accurate dietary assessment of diverse population subgroups is lacking both in Ireland and worldwide, and in consequence, there is a paucity of data examining food consumption patterns and food choice influence of differing ethnicities [43]. This in turn impacts the inclusion of such groups in nutritional research outputs and subsequent public health policy, which may lead to adverse health outcomes among minority groups [44]. Poor consideration of population subgroups when developing dietary assessment tools that are intended to be nationally representative means that many surveys conducted using such tools may not accurately depict intake of different nationalities or ethnicities living in one location. Previous work has developed assessment tools for use across different countries through food list expansion and tool translation, allowing for cross-country comparisons of dietary data [45-47]. However, these developments often cater primarily to the general populations within each country, with minor nationalities being overlooked during the tool development process. Tools that cater for minority nationalities and ethnicities are also important as data collected through national food consumption surveys do not reflect the dietary trends of countries’ nationals who have immigrated from elsewhere. When emigrating to a new country, acculturation to the host country’s dietary norms and limitations on food availability mean that the food consumption habits of immigrants inevitably change from their traditional diets [43]. However, despite the adoption of some of the host country’s dietary norms by immigrants, those from different nationalities often retain certain traditional food items in their new diet, which is particularly common among older generations [48]. Therefore, for dietary assessment to measure food intake of all subgroups of the population, including those of different ethnicities or nationalities, the usability and food lists of web-based dietary assessment tools need to be validated among population subgroups. Furthermore, subsequent diversification of existing food lists is likely to be required. Thompson et al [49] has highlighted the need to consider language, literacy, and content of food lists within dietary assessment tools that are intended to be used among diverse population groups. If used in populations outside those intended (eg, Irish adults in the case of Foodbook24), web-based assessment tools should incorporate different languages, population-specific names for food and drink items, appropriate nutrient composition source, and portion size estimates of these items [49]. In addition, future tool developments should also consider how eating habits (eg, sharing of large plates or traditional dishes) differ across cultures and allow flexibility in how portion size is assessed [7]. By translating the tool into Polish and Portuguese and expanding the food list to include a wide range of ethnic foods, Foodbook24 has been improved for accurate use among diverse populations in Ireland. Advantages of Foodbook24 cited by participants included its ease of use, convenience, and portion size quantification. Although Foodbook24 was cited as being easy to use, some population subgroups (eg, older adults) may benefit from technical support while completing web-based dietary recalls [50]. Web-based platforms such as Zoom, as used in this study, would facilitate this. Although possibly more time consuming for researchers, providing ample support to participants as needed would help improve both retention of participants and accuracy of data collected.

### Future Work

While actual measurement of intakes in diverse groups is important, the validation of dietary assessment methods for use in these diverse populations is also essential to ensure dietary patterns are accurately captured. Many comparison and validation studies do not consider differing ethnicities or nationalities within the sample population, with few assessments of other web-based dietary assessment tools worldwide obtaining a rate of participants from ethnic minorities of >10% [9,51-54]. This comparison study was not designed to examine diverse population dietary intakes but rather to test the accuracy of food intake as reported through a self-administered web-based 24-hour dietary recalls by diverse adults in Ireland. Further testing and validation of Foodbook24 among a larger pool of participants would be advantageous. The future use of Foodbook24 could help better understand the habitual diets of different nationalities living in Ireland and inform public health policies aimed at supporting specific population subgroups in meeting healthy eating guidelines and nutrient requirements. Similar tool developments across other countries are essential to understand the food intakes of minority groups globally and ensure that diverse nationalities are included in nutrition monitoring worldwide. With such developments in dietary assessment tools, research into how diets of different nationalities compare and how country of residency impacts the dietary intakes of immigrants is possible and would support public health strategies looking to address the needs of minority groups. It is recognized that inclusion of diverse population subgroups in research is challenging, but more needs to be done to support the participation of differing nationalities in research and provide insight into dietary patterns of the total population [55]. Recruitment and participant retention were major challenges faced in this research and is one that has been faced by many researchers in the past [37,56]. A scoping review by Wieland et al [57] noted community engagement to promote trust is a crucial avenue to explore when hoping to effectively recruit minority groups and future efforts should look to understand cultures and research perspectives of target groups [58].

### Strengths and Limitations

In terms from strengths of this research, participants of diverse nationalities were involved in the expansion of Foodbook24 by providing insight into their habitual eating patterns, (ie, indicating additional food items required in Foodbook24) and testing the translated versions of the tool. This is the first study to assess the accuracy of a web-based dietary assessment tool among diverse population groups in Ireland in comparison to traditional assessment methods. Self-administered and interviewer-led recalls presented here reflected the same 24-hour period. All researchers conducting interviewer-led 24-hour recalls were trained and followed a predefined protocol. The same portion size estimates and food composition database was used for both interviewer-led and self-administered recalls so that the full effect of recall method on dietary intakes could be examined.

Despite trying to achieve an equal distribution of Brazilian, Irish, and Polish participants to assess Foodbook24, low numbers were recruited across groups. Just <20% (19/104, 18.27%) of the total sample who completed the comparison study were Polish, and only 13% (4/32) of Brazilian participants were male, meaning that comparisons within and across groups must be interpreted with caution. Associations identified within Brazilian and Polish samples could be exaggerated or significant differences in intake unidentified; a larger sample size of individual groups would reduce random variation and may reduce larger SD of nutrient and food group intakes observed. Brazilian and Polish participants were unable to complete the interviewer-led recalls in their first language and this may have caused differences in reporting across methods. As with all retrospective dietary intake data collection, this study was reliant on memory of participants and is subject to recall bias. Despite every effort to facilitate participant memory (ie, food prompts and forgotten foods checklist), self-reporting remains flawed. This study focused on developing Foodbook24 for use among non-Irish nationals, who are usually younger in age. Future work may look to develop Foodbook24 among children and additional nationalities growing in Ireland. Recruitment for this study was conducted during the COVID-19 pandemic; thus, in-person engagement with community members proved particularly difficult.

### Conclusions

The expanded Foodbook24 food list is comprehensive and generally accepted by Irish, Brazilian, and Polish populations living in Ireland. Foodbook24 is now available in multiple languages, which allows users the option to select which language to record their diet in. Results reported here demonstrate that Foodbook24 is an accurate method of measuring food group and nutrient intakes in a nationally diverse sample of adults living in Ireland when compared to interviewer-led 24-hour dietary recalls. Minimal differences were observed in the food group and nutrient intakes recorded through Foodbook24 across Brazilian, Irish, and Polish groups. Validation involving a larger and more diverse sample as well as biomarker analysis could be completed in the future to further assess the tool. Overall, the expanded Foodbook24 tool allows researchers to accurately identify dietary and meal patterns across different groups living in Ireland, taking diverse language and food choice into consideration.

## Appendix

Multimedia Appendix 1 Results from individual cohorts (ie findings from Irish, Brazilian, and Polish groups separately).

## Abbreviations

CoFID: Composition of Food Integrated Database
UCD: University College Dublin

## Data Availability

The datasets generated and analyzed during this study are available in the Irish Social-Science Data Archive repository [60]. The updated Foodbook24 tool is freely available on the internet (FoodBook24). Any researcher who is interested in using Foodbook24 for their research should contact author ERG.
