# Supplementary material for: Expansion and Assessment of a Web-Based 24-Hour Dietary Recall Tool, Foodbook24, for Use Among Diverse Populations Living in Ireland: Comparative Analysis
Source: Online J Public Health Inform. 2025 Feb 7;17:e52380. doi: 10.2196/52380 (PMC11845893; doi:10.2196/52380)
Supplement: Multimedia Appendix 1 [file ojphi_v17i1e52380_app1.docx]

**Supplementary Material**

Figure 1: Participant food and portion size selection

Figure 2: Participant journey when completing 24-hour dietary recalls on Foodbook24

Table 1: Participant Demographics of adequate vs. under reporters

Table 2: Mean energy and nutrient intake of Irish sample (less under reporters)

Table 3: Mean energy and nutrients of Polish sample (less under reporters)

Table 4: Mean energy and nutrient intakes of Brazilian sample (less under reporters)

Table 5: Mean energy and nutrient intake of total sample (adequate and under reporters)

Table 6: Mean energy and nutrient intakes of Irish sample (adequate and under reporters)

Table 7: Mean energy and nutrient intakes of Polish (adequate and under reporters)

Table 8: Mean energy and nutrient intake of Brazilian sample (under and adequate reporters)

Table 9: Mean food group intakes of Irish sample (less under reporters)

Table 10: Mean food group intakes of Polish sample (less under reporters)

Table 11: Mean food group intakes of Brazilian sample (less under reporters)

Table 12: Mean food group intakes of total sample (adequate and under reporters)

Table 13: Mean food group intakes of Irish sample (adequate and under reporters)

Table 14: Mean food group intakes of Polish sample (adequate and under reporters)

Table 15: Mean food group intakes of Brazilian sample (adequate and under reporters)

Figure 3: Difference in protein (% energy) reported in the self-administered and interviewer-led recall (Irish sample)

Figure 4: Difference in carbohydrate (% energy) reported in the self-administered and interviewer-led recall (Irish sample)

Figure 5: Difference in fat (% energy) reported in the self-administered and interviewer-led recall (Irish sample)

Figure 6: Difference in sugars (grams) reported in the self-administered and interviewer-led recall (Irish sample)

Figure 7: Difference in vitamin D (ug) reported in the self-administered and interviewer-led recall (Irish sample)

Figure 8: Difference in calcium(mg) reported in the self-administered and interviewer-led recall (Irish sample)

Figure 9: Difference in protein (% energy) reported in the self-administered and interviewer-led recall (Polish sample).

Figure 10: Difference in carbohydrate (% energy) reported in the self-administered and interviewer-led recall (Polish sample)

Figure 11: Difference in fat (% energy) reported in the self-administered and interviewer-led recall (Polish sample)

Figure 12: Difference in sugars (grams) reported in the self-administered and interviewer-led recall (Polish sample)

Figure 12: Difference in vitamin D (ug) reported in the self-administered and interviewer-led recall (Polish sample)

Figure 13: Difference in calcium (mg) reported in the self-administered and interviewer-led recall (Polish sample)

Figure 14: Difference in protein (% energy) reported in the self-administered and interviewer-led recall (Brazilian sample)

Figure 15: Difference in carbohydrate (% energy) reported in the self-administered and interviewer-led recall (Brazilian sample)

Figure 16: Difference in fat (% energy) reported in the self-administered and interviewer-led recall (Brazilian sample)

Figure 17: Difference in sugar (grams) reported in the self-administered and interviewer-led recall (Brazilian sample)

Figure 18: Difference in vitamin D (ug) reported in the self-administered and interviewer-led recall (Brazilian sample)

Figure 19: Difference in calcium (mg) reported in the self-administered and interviewer-led recall (Brazilian sample)

Figure 2: Participant journey when completing 24-hour dietary recalls on Foodbook24

Table 1: Participant Demographics of adequate vs. under reporters

|  |  | **Total Sample** | **Adequate Reporters** | **Under Reporters** |
| --- | --- | --- | --- | --- |
| n (% sample) |  | 104 (100.0) | 74 (71.15) | 30 (28.85) |
|  | Irish | 53 (50.96) | 36 (67.92) | 17 (32.08) |
|  | Brazilian | 32 (30.77) | 27 (84.38) | 5 (15.63 |
|  | Polish | 19 (18.27) | 11 (57.90) | 8 (42.11) |
| Age (SD); years |  | 38.83 (14.19) | 36.54 (13.86) | 43.07 (15.21) |
| Sex  n (% sample) | Males | 29 (27.9) | 41 (75.90) | 17 (56.70) |
|  | Females | 75 (72.0) | 13 (24.10) | 13 (43.30) |
| BMI (kg/m^2^) |  | 23.51 | 23.41 | 26.39 |
| Education  n (% sample) | Level 8+ | 63 (74) | 39 (72.22) | 24 (80.00) |
|  | Other | 22 (26) | 15 (27.78) | 6 (20.00) |
| Physical Activity  n (% sample) | *≤2 weekly* | 35 (42) | 15 (27.78) | 9 (30.00) |
|  | *≥3 weekly* | 48 (58) | 39 (72.22) | 21 (70.00) |

A total of n=54 adequate reporters completed the demographic questionnaire, reported variables are based on these n=54

Table 2: Mean energy and nutrient intake of Irish sample (less under reporters)

|  | **Interviewer-led Intakes (n=38)** | **Self-admin Intakes (n=38)** | ***p* value** | **Difference** | **Correlations** |
| --- | --- | --- | --- | --- | --- |
| **Nutrients** | Mean *(SD)* | Mean *(SD)* |  | *(%)* | *r (p)* |
| Energy (kcal/day) | 2074.75 *(594.93)* | 2183.08 *(828.72)* | 0.69 | 4.96 | 0.71** |
| Protein (g/day) | 90.06 *(38.32)* | 84.18 *(32.20)* | 0.50 | -6.98 | 0.56** |
| Carbohydrate (g/day) | 244.54 *(84.90)* | 255.79 *(95.20)* | 0.67 | 4.40 | 0.77** |
| Sugars (g/day) | 105.19 *(51.11)* | 111.25 *(69.85)* | 0.85 | 5.45 | 0.87** |
| Starch (g/day) | 131.28 *(45.95)* | 133.05 *(50.21)* | 0.86 | 1.33 | 0.66** |
| Dietary Fibre (g/day) | 18.22 *(5.95)* | 18.69 *(6.67)* | 0.86 | 2.48 | 0.70** |
| Total Fat (g/day) | 84.75 *(27.52)* | 87.00 *(29.07)* | 0.75 | 2.59 | 0.67** |
| Saturated Fat (g/day) | 33.67 *(14.26)* | 32.76 *(13.41)* | 0.90 | -2.79 | 0.81** |
| Monounsaturated Fat (g/day) | 29.88 *(10.43)* | 30.94 *(12.45)* | 0.75 | 3.42 | 0.72** |
| Polyunsaturated Fat (g/day) | 6.40 *(2.48)* | 6.63 *(3.69)* | 0.78 | 3.47 | 0.46* |
| Protein (% energy) | 17.97 *(7.27)* | 15.94 *(4.83)* | 0.37 | -12.71 | 0.59** |
| Carbohydrate (% energy) | 43.85 *(6.50)* | 44.28 *(6.78)* | 0.69 | 0.97 | 0.82** |
| Total Fat (% energy) | 36.79 *(6.03)* | 36.89 *(7.81)* | 0.81 | 0.26 | 0.81** |
| Vitamin D (µg/day) | 8.00 *(10.72)* | 8.08 *(10.96)* | 0.73 | 1.07 | 0.72** |
| Vitamin E (mg/day) | 18.50 *(15.73)* | 16.86 *(12.83)* | 0.69 | -9.68 | 0.83** |
| Vitamin B6 (mg/day) | 5.80 *(12.10)* | 5.09 *(8.79)* | 0.94 | -13.91 | 0.85** |
| Vitamin B12 (µg/day) | 5.62 *(4.10)* | 9.14 *(17.06)* | 0.78 | 38.51 | 0.74** |
| Folate (mg/day) | 363.42 *(230.18)* | 365.95 *(250.03)* | 0.85 | 0.69 | 0.83** |
| Vitamin C (mg/day) | 197.79 *(228.52)* | 160.41 *(193.78)* | 0.49 | -23.30 | 0.64** |
| Calcium (mg/day) | 1002.77 *(422.65)* | 931.72 *(335.24)* | 0.56 | -7.63 | 0.79** |
| Magnesium (mg/day) | 326.97 *(108.66)* | 357.19 *(180.79)* | 0.65 | 8.46 | 0.80** |
| Phosphorus (mg/day) | 1353.82 *(346.54)* | 1400.26 *(610.89)* | 0.95 | 3.32 | 0.79** |
| Iron (mg/day) | 21.19 *(25.19)* | 21.11 *(26.81)* | 1.00 | -0.38 | 0.72** |
| Iodine (mg/day) | 169.82 *(99.64)* | 157.86 *(106.65)* | 0.38 | -7.58 | 0.81** |
| Vitamin A (µg/day) | 1032.74 *(636.06)* | 905.35 *(697.40)* | 0.20 | -14.07 | 0.72** |
| Carotene (µg/day) | 3327.44 *(2636.34)* | 3188.91 *(2926.73)* | 0.65 | -4.34 | 0.73** |

Interviewer-led= interviewer-led 24-hour dietary recall, Self-admin= self-administered 24-hour dietary recall (via Foobook24), SD=standard deviation, **= *p* value <0.001.

Table 3: Mean energy and nutrients of Polish sample (less under reporters)

|  | **Interviewer-led Intakes (n=11)** | **Self-admin Intakes (n=11)** | ***p* value** | **Difference** | **Correlations** |
| --- | --- | --- | --- | --- | --- |
| **Nutrients** | Mean *(SD)* | Mean *(SD)* |  | *(%)* | *r (p)* |
| Energy (kcal/day) | 1886.79 *(455.86)* | 2257.20 *(2257.20)* | 0.14 | 16.41 | 0.57 (0.07) |
| Protein (g/day) | 78.55 *(23.26)* | 86.41 *(20.20)* | 0.38 | 9.09 | 0.61 (0.05) |
| Carbohydrate (g/day) | 214.96 *(57.98)* | 281.49 *(100.08)* | 0.09 | 23.63 | 0.72* |
| Sugars (g/day) | 85.97 *(27.47)* | 118.68 *(70.61)* | 0.53 | 27.56 | 0.69* |
| Starch (g/day) | 118.54 *(38.51)* | 139.27 *(66.76)* | 0.82 | 14.88 | 0.83* |
| Dietary Fibre (g/day) | 16.58 *(4.00)* | 19.95 *(8.54)* | 0.49 | 16.90 | 0.46 (0.16) |
| Total Fat (g/day) | 81.92 *(25.12)* | 92.67 *(26.05)* | 0.34 | 11.59 | 0.79* |
| Saturated Fat (g/day) | 32.65 *(12.79)* | 36.54 *(16.47)* | 0.67 | 10.63 | 0.84* |
| Monounsaturated Fat (g/day) | 29.42 *(9.90)* | 30.74 *(10.84)* | 0.67 | 4.29 | 0.87** |
| Polyunsaturated Fat (g/day) | 6.68 *(2.92)* | 7.63 *(3.04)* | 0.45 | 12.46 | 0.66* |
| Protein (% energy) | 16.59 *(2.47)* | 15.65 *(2.24)* | 0.45 | -6.00 | 0.51 (0.11) |
| Carbohydrate (% energy) | 42.95 *(7.21)* | 45.93 *(6.59)* | 0.22 | 6.48 | 0.55 (0.08) |
| Total Fat (% energy) | 38.85 *(6.23)* | 37.37 *(5.87)* | 0.58 | -3.94 | 0.67* |
| Vitamin D (µg/day) | 6.29 *(5.38)* | 4.96 *(3.58)* | 0.82 | -26.85 | 0.39 (0.24) |
| Vitamin E (mg/day) | 12.34 *(4.37)* | 14.94 *(6.97)* | 0.45 | 17.40 | 0.48 (0.13) |
| Vitamin B6 (mg/day) | 6.32 *(15.08)* | 7.02 *(15.01)* | 0.34 | 9.97 | 0.51 (0.11) |
| Vitamin B12 (µg/day) | 7.37 *(3.14)* | 7.27 *(5.21)* | 0.62 | -1.26 | 0.10 (0.77) |
| Folate (mg/day) | 309.34 *(149.54)* | 291.70 *(150.07)* | 0.41 | -6.05 | 0.54 (0.09) |
| Vitamin C (mg/day) | 334.56 *(325.84)* | 266.47 *(309.26)* | 0.49 | -25.55 | 0.63* |
| Calcium (mg/day) | 1155.11 *(388.76)* | 1180.05 *(320.970* | 0.87 | 2.11 | 0.48 (0.13) |
| Magnesium (mg/day) | 389.06 *(141.51)* | 466.01 *(165.50)* | 0.34 | 16.51 | 0.47 (0.14) |
| Phosphorus (mg/day) | 1362.17 *(375.47)* | 1488.74 *(385.19)* | 0.45 | 8.50 | 0.86** |
| Iron (mg/day) | 16.43 *(7.51)* | 21.18 *(9.90)* | 0.22 | 22.42 | 0.54 (0.09) |
| Iodine (mg/day) | 153.18 *(83.69)* | 162.49 *(77.46)* | 0.72 | 5.73 | 0.04 (0.19) |
| Vitamin A (µg/day) | 1043.89 *(419.80)* | 1230.69 *(967.16)* | 0.77 | 15.18 | 0.13 (0.71) |
| Carotene (µg/day) | 4510.09 *(2381.70)* | 5974.03 *(5432.29)* | 0.77 | 24.51 | 0.16 (0.63) |

Interviewer-led= interviewer-led 24-hour dietary recall, Self-admin= self-administered 24-hour dietary recall (via Foobook24), SD=standard deviation, *= *p* value <0.05, **= *p* value <0.001.

Table 4: Mean energy and nutrient intakes of Brazilian sample (less under reporters)

|  | **Interviewer-led Intakes (n=25)** | **Self-admin Intakes (n=25)** | ***p* value** | **Difference** | **Correlations** |
| --- | --- | --- | --- | --- | --- |
| **Nutrients** | Mean *(SD)* | Mean *(SD)* |  | *(%)* | *r (p)* |
| Energy (kcal/day) | 1966.97 *(440.80)* | 1899.82 *(515.08)* | 0.47 | 0.00 | 0.48 (0.15) |
| Protein (g/day) | 89.01 *(40.14)* | 84.79 *(31.54)* | 0.82 | -4.98 | 0.50 (0.12) |
| Carbohydrate (g/day) | 232.81 *(71.38)* | 222.53 *(70.10)* | 0.53 | -4.62 | 0.52* |
| Sugars (g/day) | 90.11 *(43.31)* | 80.03 *(44.72)* | 0.27 | -12.59 | 0.71** |
| Starch (g/day) | 134.69 *(48.45)* | 127.02 *(47.16)* | 0.55 | -6.04 | 0.49 (0.13) |
| Dietary Fibre (g/day) | 18.68 *(9.15)* | 15.73 *(6.02)* | 0.39 | -18.72 | 0.61* |
| Total Fat (g/day) | 79.93 *(17.35)* | 77.60 *(24.41)* | 0.59 | -3.00 | 0.45* |
| Saturated Fat (g/day) | 30.14 *(9.16)* | 29.26 *(14.77)* | 0.43 | -3.01 | 0.68** |
| Monounsaturated Fat (g/day) | 29.31 *(7.92)* | 26.93 *(14.77)* | 0.37 | -8.84 | 0.53* |
| Polyunsaturated Fat (g/day) | 6.01 *(2.64)* | 6.79 *(3.77)* | 0.73 | 11.46 | 0.44* |
| Protein (% energy) | 17.96 *(5.64)* | 18.02 *(5.06)* | 0.88 | 0.33 | 0.60* |
| Carbohydrate (% energy) | 44.10 *(7.99)* | 44.03 *(7.16)* | 0.79 | -0.16 | 0.70** |
| Total Fat (% energy) | 37.10 (6.33) | 36.72 *(6.49)* | 0.93 | -1.03 | 0.67** |
| Vitamin D (µg/day) | 7.37 *(5.21)* | 9.37 *(8.65)* | 0.72 | 21.28 | 0.76** |
| Vitamin E (mg/day) | 12.26 *(6.91)* | 10.21 *(6.52)* | 0.27 | -20.04 | 0.69** |
| Vitamin B6 (mg/day) | 3.14 *(4.98)* | 2.00 *(1.15)* | 0.49 | -57.15 | 0.57* |
| Vitamin B12 (µg/day) | 6.17 *(3.87)* | 26.81 *(100.23)* | 0.92 | 76.97 | 0.49* |
| Folate (mg/day) | 350.02 *(251.29)* | 340.06 *(269.32)* | 0.54 | -2.93 | 0.80** |
| Vitamin C (mg/day) | 172.48 *(188.75)* | 91.73 *(80.73)* | 0.18 | -88.03 | 0.52** |
| Calcium (mg/day) | 913.87 *(307.11)* | 830.09 *(461.71)* | 0.09 | -10.09 | 0.54* |
| Magnesium (mg/day) | 315.44 *(96.56)* | 307.94 *(110.29)* | 0.64 | -2.44 | 0.64** |
| Phosphorus (mg/day) | 1353.51 *(384.95)* | 1314.37 *(476.29)* | 0.48 | -2.98 | 0.50* |
| Iron (mg/day) | 17.35 *(22.03)* | 13.68 *(6.40)* | 0.88 | -26.86 | 0.64** |
| Iodine (mg/day) | 155.89 *(94.03)* | 124.64 *(7556)* | 0.23 | -25.07 | 0.74** |
| Vitamin A (µg/day) | 1081.62 *(612.53)* | 793.14 *(551.91)* | 0.11 | -36.37 | 0.69** |
| Carotene (µg/day) | 4601.95 *(4207.48)* | 2310.34 *(2704.65)* | 0.05 | -99.19 | 0.64** |

Interviewer-led= interviewer-led 24-hour dietary recall, Self-admin= self-administered 24-hour dietary recall (via Foobook24), SD=standard deviation, *= *p* value <0.05, **= *p* value <0.001.

Table 5: Mean energy and nutrient intake of total sample (adequate and under reporters)

|  | **Interviewer-led Intakes (n=104)** | **Self-admin Intakes (n=104)** | ***p* value** | **Difference** | **Correlations** |
| --- | --- | --- | --- | --- | --- |
| **Nutrients** | Mean *(SD)* | Mean *(SD)* |  | *(%)* | *r* |
| Energy (kcal/day) | 1881.31 *(533.68)* | 1887.11 (715.16) | 0.73 | 0.31 | 0.71** |
| Protein (g/day) | 84.23 *(35.18)* | 80.25 (30.16) | 0.42 | -4.96 | 0.59** |
| Carbohydrate (g/day) | 221.51 *(77.07)* | 224.14 (91.80) | 0.90 | 1.17 | 0.71** |
| Sugars (g/day) | 91.13 *(44.38)* | 89.93 (58.85) | 0.33 | -1.33 | 0.77** |
| Starch (g/day) | 121.83 *(46.95)* | 121.20 (53.61) | 0.92 | -0.52 | 0.72** |
| Dietary Fibre (g/day) | 17.38 *(6.66)* | 16.47 (6.71) | 0.34 | -5.51 | 0.65** |
| Total Fat (g/day) | 76.75 *(24.40)* | 75.39 (28.29) | 0.58 | -1.81 | 0.69** |
| Saturated Fat (g/day) | 29.38 *(12.13)* | 28.28 (13.99) | 0.31 | -3.91 | 0.80** |
| Monounsaturated Fat (g/day) | 27.72 *(9.78)* | 26.41 (11.16) | 0.31 | -4.96 | 0.75** |
| Polyunsaturated Fat (g/day) | 6.03 *(2.81)* | 6.07 (3.67) | 0.48 | 0.66 | 0.60** |
| Protein (% energy) | 18.22 *(6.33)* | 17.76 (5.53) | 0.82 | -2.63 | 0.68** |
| Carbohydrate (% energy) | 43.88 *(7.47)* | 44.20 (7.62) | 0.60 | 0.73 | 0.75** |
| Total Fat (% energy) | 36.84 *(6.17)* | 36.45 (7.07) | 0.91 | -1.08 | 0.77** |
| Vitamin D (µg/day) | 7.52 *(8.57)* | 7.99 (9.59) | 0.61 | 5.94 | 0.72** |
| Vitamin E (mg/day) | 14.63 *(11.50)* | 14.53 (17.14) | 0.28 | -0.72 | 0.71** |
| Vitamin B6 (mg/day) | 4.41 *(9.45)* | 3.62 (7.35) | 0.50 | -21.80 | 0.69** |
| Vitamin B12 (µg/day) | 15.69 (98.08) | 16.92 (69.71) | 0.67 | 7.23 | 0.63** |
| Folate (mg/day) | 329.10 *(206.71)* | 314.32 (222.45) | 0.23 | -4.70 | 0.78** |
| Vitamin C (mg/day) | 198.81 *(248.79)* | 144.90 (188.01) | 0.16 | -37.20 | 0.66** |
| Calcium (mg/day) | 960.77 *(391.58)* | 889.02 (395.53) | 0.13 | -8.07 | 0.71** |
| Magnesium (mg/day) | 317.60 *(111.01)* | 324.08 (153.92) | 0.66 | 2.00 | 0.75** |
| Phosphorus (mg/day) | 1293.77 *(355.61)* | 1276.08 (508.77) | 0.33 | -1.39 | 0.71** |
| Iron (mg/day) | 17.02 *(19.07)* | 16.74 (17.90) | 0.74 | -1.69 | 0.68** |
| Iodine (mg/day) | 158.02 *(88.16)* | 139.80 (84.73) | 0.08 | -13.04 | 0.70** |
| Vitamin A (µg/day) | 957.91 *(580.45)* | 816.76 (652.11) | **0.02^x^** | -17.28 | 0.57** |
| Carotene (µg/day) | 3459.78 *(3139.81)* | 2871.83 (3138.92) | 0.08 | -20.47 | 0.54** |

Interviewer-led= interviewer-led 24-hour dietary recall, Self-admin= self-administered 24-hour dietary recall (via Foobook24), SD=standard deviation, ^x^= only significant for unadjusted p-values following Bonferroni correction, **= *p* value <0.001.

Table 6: Mean energy and nutrient intakes of Irish sample (adequate and under reporters)

|  | **Interviewer-led Intakes (n=53)** | **Self-admin Intakes (n=53)** | ***p* value** | **Difference** | **Correlations** |
| --- | --- | --- | --- | --- | --- |
| **Nutrients** | Mean *(SD)* | Mean *(SD)* |  | *(%)* | *r* |
| Energy (kcal/day) | 1976.12 *(565.28)* | 1969.21 *(796.78)* | 0.69 | -0.35 | 0.74** |
| Protein (g/day) | 87.03 *(36.20)* | 81.10 *(31.67)* | 0.33 | -7.31 | 0.62** |
| Carbohydrate (g/day) | 234.96 *(82.16)* | 233.82 *(95.89)* | 0.91 | -0.49 | 0.74** |
| Sugars (g/day) | 99.07 *(49.01)* | 98.40 *(64.18)* | 0.71 | -0.68 | 0.83** |
| Starch (g/day) | 127.43 *(46.02)* | 125.12 *(53.69)* | 0.83 | -1.84 | 0.74** |
| Dietary Fibre (g/day) | 17.69 *(5.83)* | 17.62 *(6.34)* | 0.86 | -0.41 | 0.74** |
| Total Fat (g/day) | 79.32 *(26.79)* | 77.07 *(29.90)* | 0.66 | -2.92 | 0.70** |
| Saturated Fat (g/day) | 30.56 *(13.44)* | 28.55 *(13.44)* | 0.52 | -7.05 | 0.81** |
| Monounsaturated Fat (g/day) | 28.51 *(10.48)* | 27.50 *(12.33)* | 0.55 | -3.66 | 0.74** |
| Polyunsaturated Fat (g/day) | 6.23 *(2.72)* | 6.00 *(3.72)* | 0.29 | -3.87 | 0.59** |
| Protein (% energy) | 18.06 *(6.73)* | 17.29 *(5.73)* | 0.68 | -4.41 | 0.65** |
| Carbohydrate (% energy) | 44.29 *(7.34)* | 44.43 *(8.09)* | 0.66 | 0.34 | 0.76** |
| Total Fat (% energy) | 36.08 *(6.42)* | 35.96 *(7.98)* | 0.89 | -0.33 | 0.79** |
| Vitamin D (µg/day) | 7.88 *(10.28)* | 7.96 *(10.93)* | 0.47 | 1.02 | 0.72** |
| Vitamin E (mg/day) | 15.81 *(13.84)* | 14.28 *(11.55)* | 0.50 | -10.71 | 0.82** |
| Vitamin B6 (mg/day) | 4.53 *(10.26)* | 4.01 *(7.51)* | 0.78 | -12.81 | 0.85** |
| Vitamin B12 (µg/day) | 23.96 *(136.77)* | 17.14 *(69.21)* | 0.78 | -39.77 | 0.75** |
| Folate (mg/day) | 338.63 *(204.08)* | 327.21 *(220.84)* | 0.47 | -3.49 | 0.82** |
| Vitamin C (mg/day) | 164.04 *(201.05)* | 133.72 *(169.31)* | 0.49 | -22.67 | 0.74** |
| Calcium (mg/day) | 986.48 *(423.09)* | 906.98 *(373.15)* | 0.40 | -8.77 | 0.77** |
| Magnesium (mg/day) | 311.70 *(99.46)* | 323.93 *(162.20)* | 0.83 | 3.77 | 0.81** |
| Phosphorus (mg/day) | 1323.90 *(332.74)* | 1312.26 *(547.84)* | 0.42 | -0.89 | 0.77** |
| Iron (mg/day) | 18.28 *(21.54)* | 17.88 *(22.96)* | 0.45 | -2.24 | 0.68** |
| Iodine (mg/day) | 159.51 *(93.85)* | 146.98 *(95.98)* | 0.38 | -8.52 | 0.80** |
| Vitamin A (µg/day) | 970.64 *(622.28)* | 812.86 *(659.17)* | 0.08 | -19.41 | 0.56** |
| Carotene (µg/day) | 3125.81 *(2787.43)* | 2717.17 *(2641.73)* | 0.50 | -15.04 | 0.62** |

Interviewer-led= interviewer-led 24-hour dietary recall, Self-admin= self-administered 24-hour dietary recall (via Foobook24), SD=standard deviation, **= *p* value <0.001.

Table 7: Mean energy and nutrient intakes of Polish (adequate and under reporters)

|  | **Interviewer-led Intakes (n=19)** | **Self-admin Intakes (n=19)** | ***p* value** | **Difference** | **Correlations** |
| --- | --- | --- | --- | --- | --- |
| **Nutrients** | Mean *(SD)* | Mean *(SD)* |  | *(%)* | *r (p)* |
| Energy (kcal/day) | 1689.77 *(450.70)* | 1893.73 *(671.26)* | 0.44 | 10.77 | 0.75** |
| Protein (g/day) | 71.08 *(23.78)* | 76.37 *(24.60)* | 0.49 | 6.93 | 0.68* |
| Carbohydrate (g/day) | 198.64 *(52.33)* | 235.63 *(96.34)* | 0.31 | 15.70 | 0.73** |
| Sugars (g/day) | 82.12 *(27.81)* | 95.99 *(60.92)* | 0.92 | 14.46 | 0.74** |
| Starch (g/day) | 106.43 *(37.21)* | 120.95 *(57.85)* | 0.51 | 12.00 | 0.77** |
| Dietary Fibre (g/day) | 16.83 *(4.59)* | 17.12 *(7.53)* | 0.57 | 1.70 | 0.25 (0.30) |
| Total Fat (g/day) | 71.35 *(24.52)* | 76.63 *(28.71)* | 0.47 | 6.88 | 0.83** |
| Saturated Fat (g/day) | 27.40 *(12.02)* | 29.43 *(15.60)* | 0.83 | 6.89 | 0.92** |
| Monounsaturated Fat (g/day) | 25.56 *(9.42)* | 25.76 *(10.61)* | 0.97 | 0.76 | 0.94** |
| Polyunsaturated Fat (g/day) | 6.08 *(2.72)* | 6.13 *(3.12)* | 0.92 | 0.88 | 0.64* |
| Protein (% energy) | 16.74 *(3.56)* | 16.51 *(3.21)* | 0.76 | -1.43 | 0.70** |
| Carbohydrate (% energy) | 44.68 *(7.05)* | 46.19 *(6.36)* | 0.39 | 3.27 | 0.70** |
| Total Fat (% energy) | 37.56 *(5.37)* | 36.45 *(5.19)* | 0.76 | -3.04 | 0.74** |
| Vitamin D (µg/day) | 7.78 *(8.22)* | 5.74 *(4.87)* | 0.55 | -35.49 | 0.51* |
| Vitamin E (mg/day) | 15.17 *(9.79)* | 14.47 *(8.43)* | 0.99 | -4.88 | 0.53* |
| Vitamin B6 (mg/day) | 6.28 *(12.66)* | 5.32 *(11.50)* | 0.65 | -18.18 | 0.54* |
| Vitamin B12 (µg/day) | 7.66 *(4.64)* | 6.70 *(4.55)* | 0.57 | -14.28 | 0.45 (0.06) |
| Folate (mg/day) | 318.06 *(163.90)* | 288.57 *(169.02)* | 0.37 | -10.22 | 0.55* |
| Vitamin C (mg/day) | 362.59 *(386.92)* | 248.41 *(300.67)* | 0.41 | -45.97 | 0.63* |
| Calcium (mg/day) | 1031.76 *(421.75)* | 985.78 *(371.14)* | 0.80 | -4.66 | 0.70** |
| Magnesium (mg/day) | 361.87 *(152.79)* | 383.82 *(178.09)* | 0.87 | 5.72 | 0.71** |
| Phosphorus (mg/day) | 1212.88 *(373.28)* | 1242.07 *(458.52)* | 0.87 | 2.35 | 0.82** |
| Iron (mg/day) | 14.89 *(6.82)* | 17.15 *(9.79)* | 0.57 | 13.19 | 0.72** |
| Iodine (mg/day) | 152.98 *(75.76)* | 149.20 *(70.09)* | 0.92 | -2.53 | 0.56* |
| Vitamin A (µg/day) | 863.70 *(413.05)* | 959.31 *(809.92)* | 0.51 | 9.97 | 0.46 (0.05) |
| Carotene (µg/day) | 3400.57 *(2414.50)* | 4539.24 *(4567.93)* | 0.90 | 25.09 | 0.54* |

Interviewer-led= interviewer-led 24-hour dietary recall, Self-admin= self-administered 24-hour dietary recall (via Foobook24), SD=standard deviation, *= *p* value <0.05, **= *p* value <0.001.

Table 8: Mean energy and nutrient intake of Brazilian sample (under and adequate reporters)

Interviewer-led= interviewer-led 24-hour dietary recall, Self-admin= self-administered 24-hour dietary recall (via Foobook24), SD=standard deviation, ^x^= only significant for unadjusted p-values following Bonferroni correction, **= *p* value <0.001.

|  | **Interviewer-led Intakes (n=32)** | **Self-admin Intakes (n=32)** | ***p* value** | **Difference** | **Correlations** |
| --- | --- | --- | --- | --- | --- |
| **Nutrients** | Mean *(SD)* | Mean *(SD)* |  | *(%)* | *r* |
| Energy (kcal/day) | 1836.60 *(501.42)* | 1742.69 *(578.48)* | 0.39 | -5.39 | 0.63** |
| Protein (g/day) | 87.50 *(38.24)* | 81.18 *(31.28)* | 0.51 | -7.78 | 0.49** |
| Carbohydrate (g/day) | 212.52 *(78.20)* | 200.55 *(79.51)* | 0.55 | -5.97 | 0.70** |
| Sugars (g/day) | 83.09 *(42.90)* | 71.75 *(43.81)* | 0.18 | -15.80 | 0.75** |
| Starch (g/day) | 121.69 *(52.81)* | 114.63 *(51.90)* | 0.67 | -6.16 | 0.67** |
| Dietary Fibre (g/day) | 17.19 *(8.89)* | 14.12 *(6.40)* | 0.22 | -21.74 | 0.68** |
| Total Fat (g/day) | 75.68 *(19.71)* | 71.77 *(25.66)* | 0.43 | -5.45 | 0.60** |
| Saturated Fat (g/day) | 28.58 *(9.74)* | 27.10 *(14.29)* | 0.26 | -5.43 | 0.74** |
| Monounsaturated Fat (g/day) | 27.69 *(8.82)* | 24.94 *(9.36)* | 0.27 | -11.03 | 0.63** |
| Polyunsaturated Fat (g/day) | 5.65 *(3.07)* | 6.15 *(4.01)* | 0.87 | 8.08 | 0.62** |
| Protein (% energy) | 19.42 *(6.87)* | 19.31 *(6.09)* | 0.91 | -0.54 | 0.66** |
| Carbohydrate (% energy) | 42.69 *(8.01)* | 42.57 *(7.38)* | 0.82 | -0.26 | 0.68** |
| Total Fat (% energy) | 37.71 *(6.22)* | 37.29 *(6.51)* | 0.91 | -1.13 | 0.72** |
| Vitamin D (µg/day) | 6.74 *(4.99)* | 9.42 *(9.26)* | 0.56 | 28.53 | 0.75** |
| Vitamin E (mg/day) | 12.28 *(7.22)* | 14.98 *(26.93)* | 0.36 | 18.02 | 0.69** |
| Vitamin B6 (mg/day) | 3.06 *(4.54)* | 1.91 *(1.14)* | 0.25 | -60.35 | 0.47* |
| Vitamin B12 (µg/day) | 6.48 *(4.11)* | 22.79 *(90.04)* | 0.88 | 71.56 | 0.46* |
| Folate (mg/day) | 319.56 *(237.98)* | 308.07 *(256.55)* | 0.49 | -3.73 | 0.79** |
| Vitamin C (mg/day) | 157.87 *(172.36)* | 100.57 *(84.92)* | 0.27 | -56.97 | 0.52** |
| Calcium (mg/day) | 873.30 *(303.45)* | 799.01 *(439.35)* | 0.11 | -9.30 | 0.58** |
| Magnesium (mg/day) | 300.55 *(95.61)* | 287.74 *(110.86)* | 0.50 | -4.45 | 0.66** |
| Phosphorus (mg/day) | 1291.83 *(385.60)* | 1235.07 *(478.46)* | 0.38 | -4.60 | 0.57** |
| Iron (mg/day) | 16.17 *(19.96)* | 14.53 *(10.53)* | 0.96 | -11.32 | 0.64** |
| Iodine (mg/day) | 158.57 *(87.70)* | 121.75 *(70.79)* | 0.06 | -30.24 | 0.66** |
| Vitamin A (µg/day) | 993.88 *(603.93)* | 736.05 *(528.68)* | 0.10 | -35.03 | 0.67** |
| Carotene (µg/day) | 4067.04 *(3996.58)* | 2114.29 *(2547.07)* | **0.04^x^** | -92.36 | 0.60** |

Table 9: Mean food group intakes of Irish sample (less under reporters)

|  | **Interviewer-led Intakes (n=38)** | **Self-admin Intakes (n-38)** | ***p* value** | **Difference** | **Correlations** |
| --- | --- | --- | --- | --- | --- |
| **Food Group *(g/day)*** | Mean *(SD)* | Mean *(SD)* |  | *(%)* | *r (p)* |
| Beverages^+^ | 1121.24 *(1320.27)* | 1159.171 *(1875.42)* | 0.55 | 3.27 | 0.93** |
| Biscuits, Cakes and Buns | 49.13 *(37.51)* | 51.54 *(39.06)* | 0.96 | 4.69 | 0.87** |
| Breads, Rolls and Scones | 86.20 *(49.83)* | 71.27 *(44.17)* | 0.24 | -20.94 | 0.72** |
| Breakfast Cereals | 102.54 *(88.80)* | 103.02 *(91.13)* | 0.93 | 0.47 | 0.98** |
| Butter, Spreads and Oils | 12.41 *(8.71)* | 12.46 *(8.44)* | 0.94 | 0.45 | 0.80** |
| Cheese | 24.50 *(14.43)* | 20.69 *(14.10)* | 0.44 | -18.39 | 0.69* |
| Creams, Ice-creams and Desserts | 81.27 *(63.28)* | 60.59 *(59.70)* | 0.42 | -34.13 | 0.98** |
| Egg and Egg Dishes | 85.46 *(77.50)* | 73.45 *(33.06)* | 1.00 | -16.34 | 0.82* |
| Fish and Fish Dishes | 47.44 *(25.84)* | 66.94 *(63.74)* | 0.82 | 29.13 | 0.26 (0.51) |
| Fruit and Fruit Juices | 260.33 *(267.41)* | 251.80 *(199.89)* | 0.72 | -3.39 | 0.88** |
| Grains, Rice, Pasta and Savouries | 157.98 *(103.05)* | 172.51 *(102.77)* | 0.55 | 8.42 | 0.60** |
| Meat and Meat Products | 164.92 *(92.71)* | 177.10 *(90.38)* | 0.62 | 6.87 | 0.64** |
| Milk and Yoghurts | 151.16 *(117.99)* | 120.98 *(85.19)* | 0.56 | -24.95 | 0.64** |
| Nuts, Herbs and Seeds | 8.33 *(6.95)* | 13.61 *(12.92)* | 0.23 | 38.75 | 0.39 (0.13) |
| Potatoes and Potato Dishes | 96.94 *(45.26)* | 107.66 *(55.98)* | 0.56 | 9.96 | 0.73** |
| Soups, Sauces and Miscellaneous | 67.95 *(85.78)* | 67.79 *(70.84)* | 0.68 | -0.24 | 0.46* |
| Sugars, Confectionary, Preserves | 41.74 *(30.96)* | 48.79 *(39.37)* | 0.58 | 14.45 | 0.79** |
| Vegetables and Vegetable Dishes | 189.72 *(113.38)* | 170.58 *(114.79)* | 0.35 | -11.22 | 0.73** |

Interviewer-led= interviewer-led 24-hour dietary recall, Self-admin= self-administered 24-hour dietary recall (via Foobook24), SD=standard deviation, += water removed from Beverage’s food category, *=*p* value <0.01, **= *p* value <0.001.

Table 10: Mean food group intakes of Polish sample (less under reporters)

|  | **Interviewer-led Intakes (n=11)** | **Self-admin Intakes (n=11)** | ***p* value** | **Difference** | **Correlations** |
| --- | --- | --- | --- | --- | --- |
| **Food Group *(g/day)*** | Mean *(SD)* | Mean *(SD)* |  | *(%)* | *r (p)* |
| Beverages^+^ | 1079.77 *(388.36)* | 941.59 *(502.99)* | 0.22 | -14.68 | 0.72* |
| Biscuits, Cakes and Buns | 28.50 *(23.60)* | 63.56 *(84.96)* | 0.23 | 55.16 | 0.90* |
| Breads, Rolls and Scones | 52.50 *(26.88)* | 79.44 *(62.49)* | 0.54 | 33.92 | 0.63 (0.07) |
| Breakfast Cereals | 166.57 *(82.70)* | 166.79 *(87.31)* | 0.95 | 0.13 | 0.92* |
| Butter, Spreads and Oils | 10.14 *(3.76)* | 13.93 *(9.08)* | 0.70 | 27.18 | 0.36 (0.43) |
| Cheese | 31.40 *(19.44)* | 24.13 *(10.37)* | 0.65 | -30.16 | 0.77* |
| Creams, Ice-creams and Desserts | 27.25 *(8.13)* | 19.00 *(3.54)* | 0.22 | -43.42 | -1.00 |
| Egg and Egg Dishes | 189.90 *(142.13)* | 180.70 *(220.57)* | 0.83 | -5.09 | 0.98* |
| Fish and Fish Dishes | 85.50 *(85.36)* | 79.63 *(73.09)* | 0.89 | -7.38 | 1.00 |
| Fruit and Fruit Juices | 360.75 *(199.93)* | 341.75 *(233.39)* | 0.71 | -5.56 | 0.41 (0.24) |
| Grains, Rice, Pasta and Savouries | 149.36 *(102.59)* | 157.50 *(104.27)* | 0.83 | 5.17 | 0.77* |
| Meat and Meat Products | 126.00 *(75.15)* | 123.25 *(97.86)* | 0.73 | -2.23 | 0.79* |
| Milk and Yoghurts | 153.71 *(116.51)* | 209.29 *(148.77)* | 0.56 | 26.55 | 0.30 (0.62) |
| Nuts, Herbs and Seeds | 11.44 *(3.80)* | 19.36 *(8.91)* | 0.06 | 40.91 | 0.49 (0.27) |
| Potatoes and Potato Dishes | 88.00 *(47.90)* | 96.25 *(54.63)* | 0.88 | 8.57 | 1.00 |
| Soups, Sauces and Miscellaneous | 212.55 *(140.17)* | 211.75 *(183.63)* | 0.87 | -0.38 | 0.78* |
| Sugars, Confectionary, Preserves | 36.92 *(21.73)* | 43.89 *(33.61)* | 0.90 | 15.89 | 0.82* |
| Vegetables and Vegetable Dishes | 301.55 *(139.66)* | 302.15 *(201.21)* | 1.00 | 0.20 | 0.87* |

Interviewer-led= interviewer-led 24-hour dietary recall, Self-admin= self-administered 24-hour dietary recall (via Foobook24), SD=standard deviation, += water removed from Beverage’s food category, *=*p* value <0.05, **= *p* value <0.001.

Table 11: Mean food group intakes of Brazilian sample (less under reporters)

|  | **Interviewer-led Intakes (n=25)** | **Self-admin Intakes (n=25)** | ***p* value** | **Difference** | **Correlations** |
| --- | --- | --- | --- | --- | --- |
| **Food Group *(g/day)*** | Mean *(SD)* | Mean *(SD)* |  | *(%)* | *r (p)* |
| Beverages^+^ | 705.82 *(401.62)* | 561.56 *(361.59)* | 0.10 | -25.69 | 0.74** |
| Biscuits, Cakes and Buns | 53.62 *(36.88)* | 45.43 *(40.55)* | 0.32 | -18.01 | 0.58* |
| Breads, Rolls and Scones | 67.02 *(32.86)* | 52.62 *(30.83)* | 0.20 | -27.38 | 0.51* |
| Breakfast Cereals | 70.42 *(60.71)* | 72.31 *(57.34)* | 0.94 | 2.61 | 0.91** |
| Butter, Spreads and Oils | 9.72 *(4.44)* | 7.14 *(3.96)* | **0.05^x^** | -36.10 | -0.51 (0.84) |
| Cheese | 26.78 *(29.04)* | 19.57 *(15.05)* | 0.55 | -36.81 | 0.36 (0.21) |
| Creams, Ice-creams and Desserts | 53.33 *(55.69)* | 43.56 *(17.74)* | 0.41 | -22.43 | 0.20 (0.70) |
| Egg and Egg Dishes | 98.83 *(40.53)* | 102.96 *(49.64)* | 1.00 | 4.01 | 0.25 (0.44) |
| Fish and Fish Dishes | 66.73 *(64.61)* | 76.36 *(69.02)* | 0.69 | 12.62 | 0.97** |
| Fruit and Fruit Juices | 247.59 *(249.96)* | 254.50 *(242.85)* | 0.91 | 2.72 | 0.91** |
| Grains, Rice, Pasta and Savouries | 175.27 *(110.21)* | 187.28 *(98.37)* | 0.57 | 6.41 | 0.47 (0.39) |
| Meat and Meat Products | 155.04 *(63.08)* | 159.84 *(107.16)* | 0.92 | 3.01 | 0.63* |
| Milk and Yoghurts | 114.00 *(91.75)* | 159.54 *(212.26)* | 0.86 | 28.55 | 0.78* |
| Nuts, Herbs and Seeds | 9.76 *(8.44)* | 11.79 *(6.92)* | 0.25 | 17.20 | 0.44 (0.15) |
| Potatoes and Potato Dishes | 115.65 *(63.08)* | 125.18 *(87.85)* | 0.98 | 7.61 | 0.29 (0.36) |
| Soups, Sauces and Miscellaneous | 89.95 *(96.63)* | 90.92 *(87.82)* | 0.84 | 1.06 | 0.86** |
| Sugars, Confectionary, Preserves | 32.56 *(44.82)* | 26.11 *(37.64)* | 0.37 | -24.70 | 0.89** |
| Vegetables and Vegetable Dishes | 174.77 *(124.66)* | 150.85 *(147.57)* | 0.34 | -15.85 | 0.81** |

Interviewer-led= interviewer-led 24-hour dietary recall, Self-admin= self-administered 24-hour dietary recall (via Foobook24), SD=standard deviation, ^x^= only significant for unadjusted p-values following Bonferroni correction, += water removed from Beverage’s food category, *=*p* value <0.05, **= *p* value <0.001.

Table 12: Mean food group intakes of total sample (adequate and under reporters)

|  | **Interviewer-led Intakes (n=104)** | **Self-admin Intakes (n=104)** | ***p* value** | **Difference** | **Correlations** |
| --- | --- | --- | --- | --- | --- |
| **Food Group *(g/day)*** | Mean *(SD)* | Mean *(SD)* |  | *(%)* | *r (p)* |
| Beverages | 2561.29 *(1452.84)* | 1541.54 *(1548.88)* | **<0.001^xx^** | -28.89 | 0.34** |
| Biscuits, Cakes and Buns | 46.65 *(33.32)* | 48.72 *(44.10)* | 0.72 | 4.87 | 0.73** |
| Breads, Rolls and Scones | 75.03 *(49.15)* | 65.04 *(49.22)* | 0.07 | -1.65 | 0.64** |
| Breakfast Cereals | 96.53 *(81.15)* | 100.77 *(81.44)* | 0.76 | 1.88 | 0.93** |
| Butter, Spreads and Oils | 10.88 *(6.71)* | 9.86 *(7.18)* | 0.14 | 3.69 | 0.50** |
| Cheese | 26.82 (20.60) | 22.19 *(15.15)* | 0.18 | -31.86 | 0.55** |
| Creams, Ice-creams and Desserts | 56.56 *(53.43)* | 49.80 *(44.35)* | **<0.001^xx^** | -1.60 | 0.72** |
| Egg and Egg Dishes | 98.99 *(77.11)* | 90.45 *(87.42)* | **<0.001^xx^** | -48.18 | 0.73** |
| Fish and Fish Dishes | 66.04 *(56.14)* | 80.86 *(66.48)* | **0.03^x^** | 15.54 | 0.68** |
| Fruit and Fruit Juices | 262.70 *(250.81)* | 251.01 *(207.29)* | **<0.001^xx^** | -9.33 | 0.88** |
| Grains, Rice, Pasta and Savouries | 148.95 *(98.15)* | 169.15 *(97.05)* | **0.00^xx^** | 14.82 | 0.55** |
| Meat and Meat Products | 159.96 *(94.77)* | 162. 51 *(100.33)* | 0.56 | 11.06 | 0.65** |
| Milk and Yoghurts | 147.94 *(123.86)* | 155.69 *(138.38)* | 0.08 | -13.73 | 0.66** |
| Nuts, Herbs and Seeds | 9.12 *(7.81)* | 14.09 *(10.71)* | **<0.001^xx^** | 30.93 | 0.55** |
| Potatoes and Potato Dishes | 98.36 *(50.20)* | 106.23 *(61.60)* | **<0.001^xx^** | 7.27 | 0.64** |
| Soups, Sauces and Miscellaneous | 93.55 *(108.31)* | 93.91 *(107.14)* | **0.00^xx^** | -58.06 | 0.77** |
| Sugars, Confectionary, Preserves | 36.49 *(33.58)* | 38.63 *(36.49)* | **0.02^xx^** | 18.58 | 0.90** |
| Vegetables and Vegetable Dishes | 181.09 *(121.95)* | 160.47 *(134.80)* | **<0.001^xx^** | -16.97 | 0.81** |

Interviewer-led= interviewer-led 24-hour dietary recall, Self-admin= self-administered 24-hour dietary recall (via Foobook24), SD=standard deviation, ^x^= only significant for unadjusted p-values following Bonferroni correction, ^xx^= significant for adjusted and unadjusted p-values following Bonferroni correction, *=*p* value <0.05, **= *p* value <0.001.

Table 13: Mean food group intakes of Irish sample (adequate and under reporters)

|  | **Interviewer-led Intakes (n=53)** | **Self-admin Intakes (n=53)** | ***p* value** | **Difference** | **Correlations** |
| --- | --- | --- | --- | --- | --- |
| **Food Group *(g/day)*** | Mean *(SD)* | Mean *(SD)* |  | *(%)* | *r (p)* |
| Beverages | 2879.64 *(1700.75)* | 1987.23 *(1847.56)* | **<0.001^xx^** | -44.91 | 0.54* |
| Biscuits, Cakes and Buns | 48.48 *(34.54)* | 49.04 *(36.76)* | 0.78 | 1.13 | 0.83* |
| Breads, Rolls and Scones | 90.15 *(57.13)* | 73.81 *(55.94)* | 0.07 | -22.13 | 0.75* |
| Breakfast Cereals | 92.76 *(86.29)* | 98.38 *(88.43)* | 0.81 | 5.72 | 0.96* |
| Butter, Spreads and Oils | 12.32 *(8.05)* | 11.30 *(7.83)* | 0.54 | -9.03 | 0.71* |
| Cheese | 22.82 *(12.88)* | 20.34 *(12.24)* | 0.47 | -12.19 | 0.67* |
| Creams, Ice-creams and Desserts | 66.01 *(56.27)* | 55.67 *(52.70)* | 0.57 | -18.58 | 0.97* |
| Egg and Egg Dishes | 81.61 *(70.55)* | 66.81 *(41.74)* | 0.58 | -22.15 | 0.77* |
| Fish and Fish Dishes | 50.50 *(28.10)* | 78.19 *(62.63)* | 0.27 | 35.42 | 0.34 (0.17) |
| Fruit and Fruit Juices | 255.20 *(249.22)* | 240.27 *(187.99)* | 0.91 | -6.21 | 0.87* |
| Grains, Rice, Pasta and Savouries | 148.36 *(97.24)* | 174.86 *(98.18)* | 0.18 | 15.15 | 0.58* |
| Meat and Meat Products | 171.17 *(94.93)* | 179.85 *(96.53)* | 0.65 | 4.83 | 0.65* |
| Milk and Yoghurts | 150.26 *(120.81)* | 130.09 *(89.95)* | 0.77 | -15.51 | 0.70* |
| Nuts, Herbs and Seeds | 7.29 *(6.49)* | 13.20 *(12.78)* | 0.10 | 44.77 | 0.45 (0.05) |
| Potatoes and Potato Dishes | 99.22 *(47.00)* | 106.07 *(53.52)* | 0.65 | 6.46 | 0.76* |
| Soups, Sauces and Miscellaneous | 56.67 *(75.98)* | 59.19 *(67.75)* | 0.71 | 4.25 | 0.47* |
| Sugars, Confectionary, Preserves | 38.83 *(29.82)* | 44.82 *(37.27)* | 0.58 | 13.36 | 0.84* |
| Vegetables and Vegetable Dishes | 175.79 *(111.35)* | 154.82 *(108.70)* | 0.26 | -13.54 | 0.78* |

| Interviewer-led= interviewer-led 24-hour dietary recall, Self-admin= self-administered 24-hour dietary recall (via Foobook24), SD=standard deviation, ^xx^= significant for adjusted and unadjusted p-values following Bonferroni correction, *=*p* value <0.05, **= *p* value <0.001. |  |  |  |
| --- | --- | --- | --- |

Table 14: Mean food group intakes of Polish sample (adequate and under reporters)

|  | **Interviewer-led Intakes (n=19)** | **Self-admin Intakes (n=19)** | ***p* value** | **Difference** | **Correlations** |
| --- | --- | --- | --- | --- | --- |
| **Food Group *(g/day)*** | Mean *(SD)* | Mean *(SD)* |  | *(%)* | *r (p)* |
| Beverages | 1639.53 *(647.99)* | 1326.82 *(829.56)* | 0.08 | -23.57 | 0.51* |
| Biscuits, Cakes and Buns | 28.36 *(21.25)* | 54.61 *(73.24)* | 0.29 | 48.06 | 0.70* |
| Breads, Rolls and Scones | 56.79 *(29.87)* | 66.41 *(50.70)* | 0.92 | 14.48 | 0.47 (0.06) |
| Breakfast Cereals | 141.45 *(76.82)* | 137.32 *(81.30)* | 0.79 | -3.01 | 0.86* |
| Butter, Spreads and Oils | 9.50 *(4.49)* | 11.27 *(8.01)* | 0.86 | 15.71 | 0.38 (0.22) |
| Cheese | 35.03 *(21.98)* | 30.46 *(20.67)* | 0.57 | -15.00 | 0.79 |
| Creams, Ice-creams and Desserts | 27.25 *(8.13)* | 19.00 *(3.54)* | 0.22 | -43.42 |  |
| Egg and Egg Dishes | 137.39 *(119.10)* | 125.22 *(169.35)* | 0.82 | -9.72 | 0.72* |
| Fish and Fish Dishes | 83.20 *(74.10)* | 78.50 *(63.35)* | 0.83 | -5.99 | 0.98* |
| Fruit and Fruit Juices | 346.76 *(281.08)* | 327.50 *(228.48)* | 0.91 | -5.88 | 0.66* |
| Grains, Rice, Pasta and Savouries | 132.50 *(88.66)* | 159.79 *(100.20)* | 0.33 | 17.08 | 0.48 (0.05) |
| Meat and Meat Products | 140.46 *(95.27)* | 140.35 *(110.18)* | 0.87 | -0.08 | 0.74 |
| Milk and Yoghurts | 162.86 *(120.67)* | 200.73 *(161.68)* | 0.74 | 18.86 | 0.49 (0.18) |
| Nuts, Herbs and Seeds | 13.18 *(8.85)* | 19.05 *(9.68)* | 0.11 | 30.80 | 0.62* |
| Potatoes and Potato Dishes | 75.19 *(38.41)* | 81.13 *(44.36)* | 0.92 | 7.32 |  |
| Soups, Sauces and Miscellaneous | 190.78 *(135.24)* | 171.89 *(157.75)* | 0.59 | -10.99 | 0.93* |
| Sugars, Confectionary, Preserves | 36.42 *(28.05)* | 41.07 *(31.74)* | 0.89 | 11.31 | 0.90* |
| Vegetables and Vegetable Dishes | 219.25 *(151.05)* | 214.00 *(187.61)* | 0.70 | -2.45 | 0.88* |

Interviewer-led= interviewer-led 24-hour dietary recall, Self-admin= self-administered 24-hour dietary recall (via Foobook24), SD=standard deviation, += water removed from Beverage’s food category, *=*p* value <0.05, **= *p* value <0.001.

Table 15: Mean food group intakes of Brazilian sample (adequate and under reporters)

|  | **Interviewer-led Intakes (n=32)** | **Self-admin Intakes (n=32)** | ***p* value** | **Difference** | **Correlations** |
| --- | --- | --- | --- | --- | --- |
| **Food Group *(g/day)*** | Mean *(SD)* | Mean *(SD)* |  | *(%)* | *r (p)* |
| Beverages | 2581.97 *(1092.36)* | 911.18 *(1018.04)* | **<0.001^xx^** | -183.37 | 0.01 (0.94) |
| Biscuits, Cakes and Buns | 52.13 *(34.08)* | 44.18 *(38.11)* | 0.19 | -17.99 | 0.42 (0.18) |
| Breads, Rolls and Scones | 59.62 *(33.45)* | 49.02 *(29.71)* | 0.29 | -21.61 | 0.16 (0.48) |
| Breakfast Cereals | 70.68 *(58.34)* | 77.71 *(58.69)* | 0.75 | 9.05 | 0.10 (0.81) |
| Butter, Spreads and Oils | 9.20 *(4.42)* | 6.54 *(3.96)* | **0.02^xx^** | -40.77 | 0.09 (0.74) |
| Cheese | 26.86 *(26.47)* | 19.11 *(13.25)* | 0.42 | -40.55 | 0.46 (0.07) |
| Creams, Ice-creams and Desserts | 46.41 *(52.25)* | 43.56 *(17.74)* | 0.22 | -6.53 | 1.00 |
| Egg and Egg Dishes | 97.97 *(44.44)* | 97.97 *(48.19)* | 0.95 | 0.00 | 0.33 (0.47) |
| Fish and Fish Dishes | 78.16 *(71.27)* | 84.83 *(75.79)* | 0.91 | 7.87 | 0.43 (0.19) |
| Fruit and Fruit Juices | 225.57 *(231.72)* | 229.06 *(225.34)* | 0.98 | 1.52 | 0.21 (0.31) |
| Grains, Rice, Pasta and Savouries | 161.41 *(107.27)* | 168.19 *(98.07)* | 0.71 | 4.03 | 0.01 (0.97) |
| Meat and Meat Products | 150.30 *(95.28)* | 147.33 *(101.49)* | 0.70 | -2.01 | 0.28 (0.19) |
| Milk and Yoghurts | 134.53 *(136.81)* | 184.43 *(202.33)* | 0.62 | 27.06 | 0.34 (0.42) |
| Nuts, Herbs and Seeds | 8.93 *(8.12)* | 11.62 *(6.66)* | 0.11 | 23.10 | 0.50 (0.12) |
| Potatoes and Potato Dishes | 108.22 *(59.96)* | 118.35 *(80.72)* | 0.91 | 8.56 | 0.93** |
| Soups, Sauces and Miscellaneous | 91.57 *(98.48)* | 95.68 *(88.75)* | 0.83 | 4.29 | 0.08 (0.76) |
| Sugars, Confectionary, Preserves | 32.23 *(43.04)* | 26.55 *(36.31)* | 0.45 | -21.38 | 0.05 (0.85) |
| Vegetables and Vegetable Dishes | 167.37 *(120.18)* | 138.80 *(136.68)* | 0.26 | -20.58 | 0.31 (0.10) |

Interviewer-led= interviewer-led 24-hour dietary recall, Self-admin= self-administered 24-hour dietary recall (via Foobook24), SD=standard deviation, ^xx^= significant for adjusted and unadjusted p-values following Bonferroni correction, *=*p* value <0.01, **= *p* value <0.001.


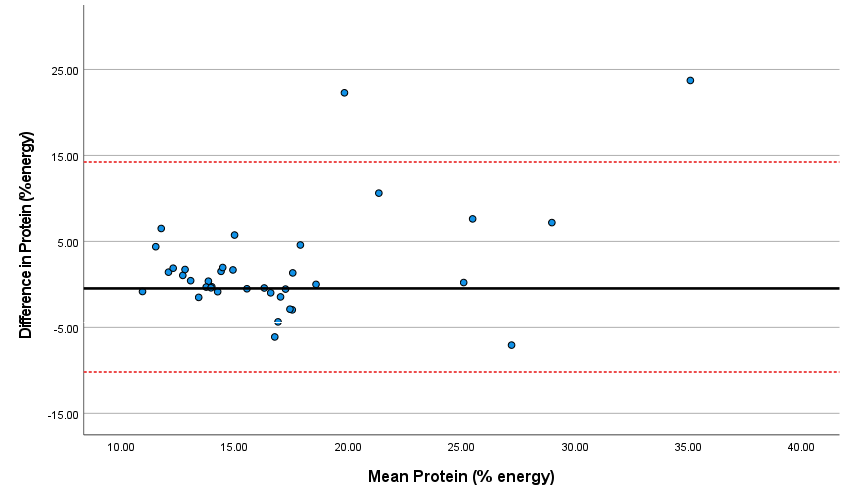


Figure 3. *Difference in protein (% energy) reported in the self-administered and interviewer-led recall (Irish sample).*


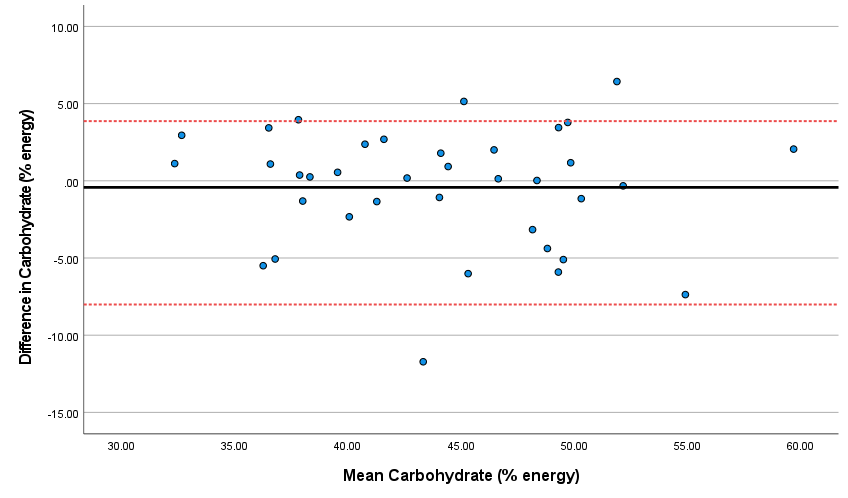


Figure 4. *Difference in carbohydrate (% energy) reported in the self-administered and interviewer-led recall (Irish sample).*


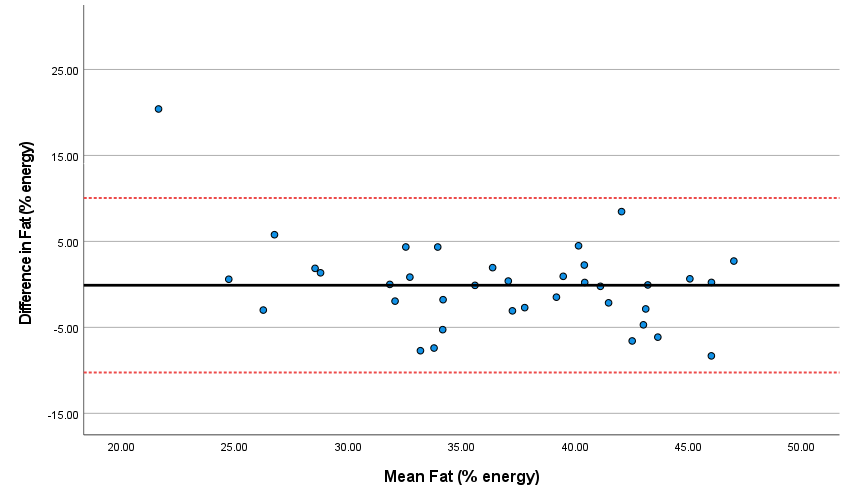


Figure 5. *Difference in fat (% energy) reported in the self-administered and interviewer-led recall (Irish sample).*


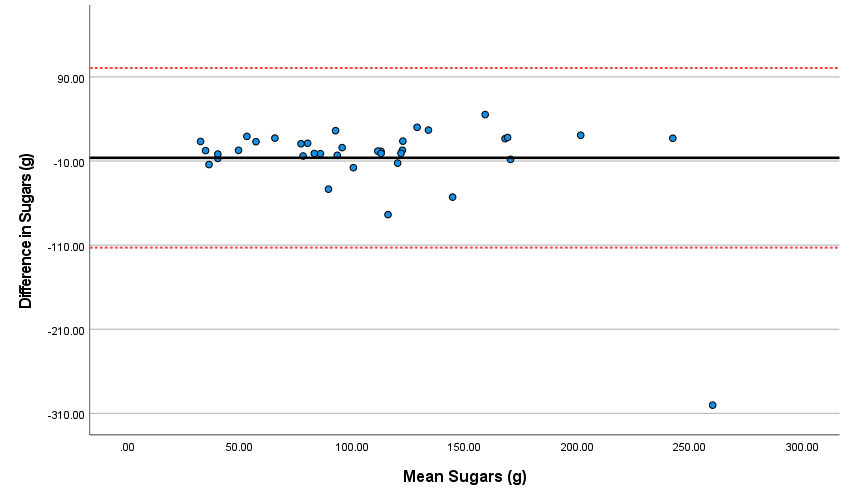


Figure 6. *Difference in sugars (grams) reported in the self-administered and interviewer-led recall (Irish sample).*


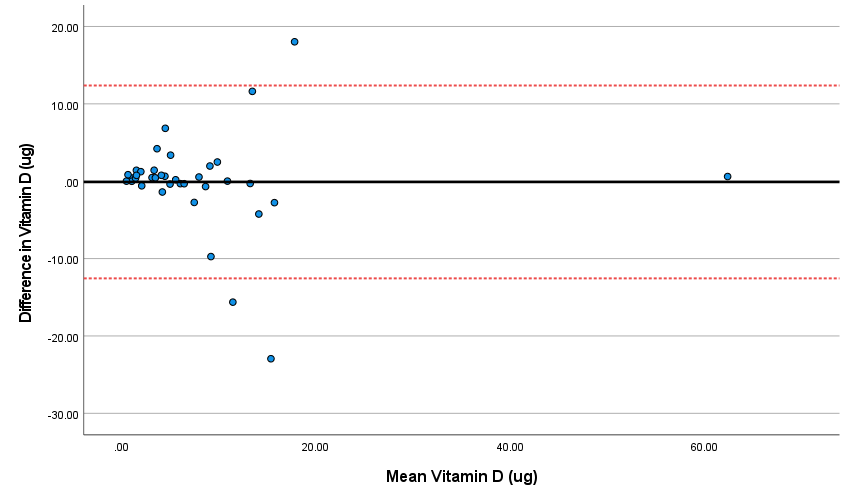


Figure 7. *Difference in vitamin D (ug) reported in the self-administered and interviewer-led recall (Irish sample).*


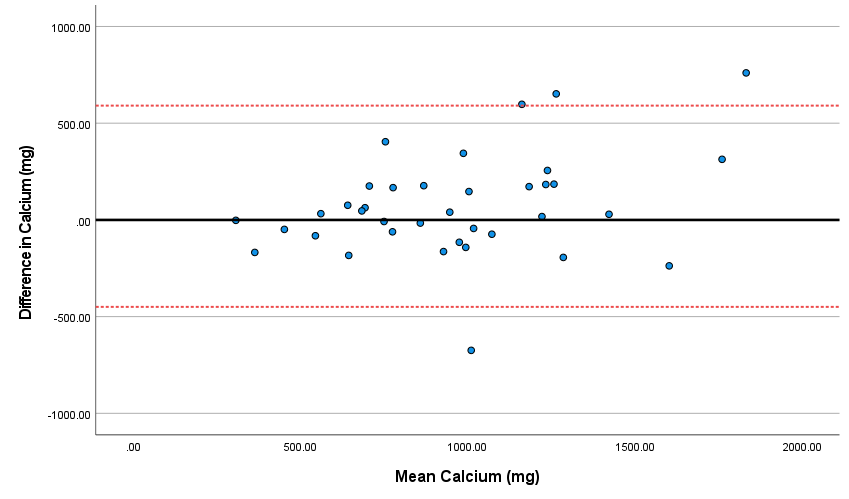


Figure 8. *Difference in calcium(mg) reported in the self-administered and interviewer-led recall (Irish sample).*


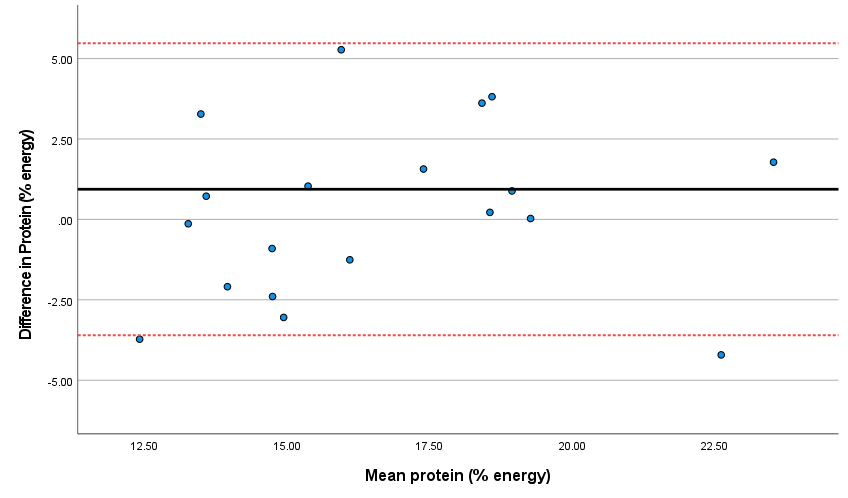


Figure 9. *Difference in protein (% energy) reported in the self-administered and interviewer-led recall (Polish sample).*


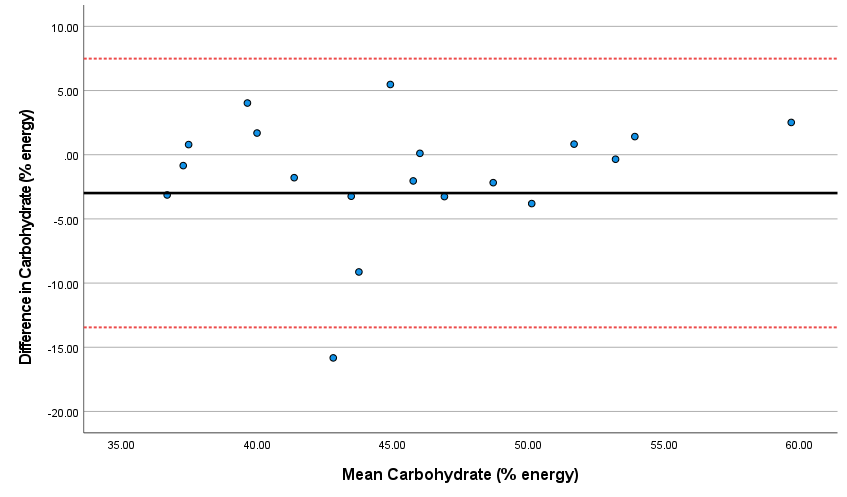


Figure 10. *Difference in carbohydrate (% energy) reported in the self-administered and interviewer-led recall (Polish sample).*


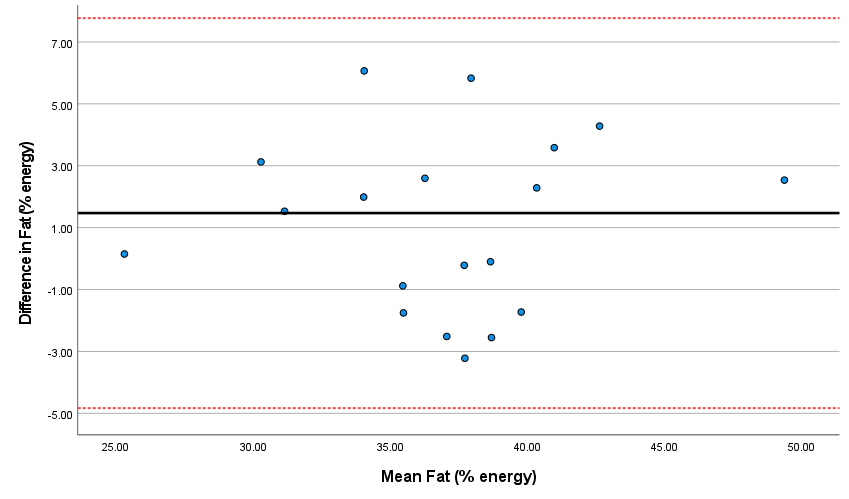


Figure 11. *Difference in fat (% energy) reported in the self-administered and interviewer-led recall (Polish sample).*


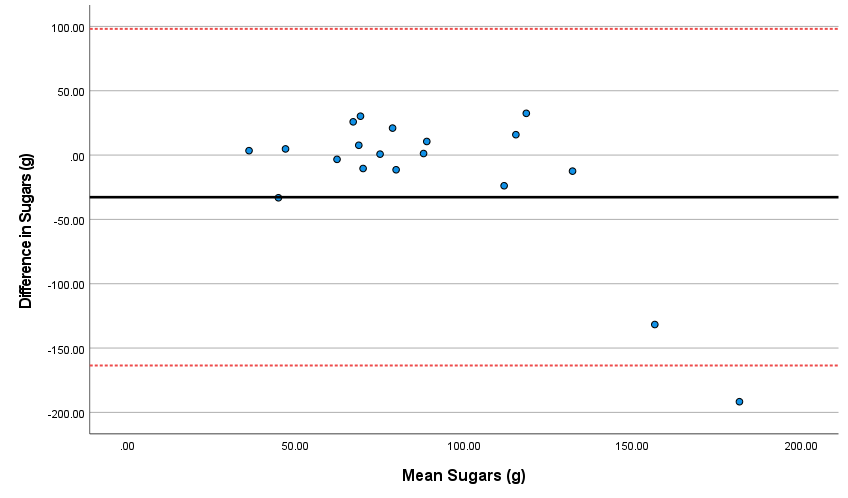


Figure 12. *Difference in sugars (grams) reported in the self-administered and interviewer-led recall (Polish sample).*


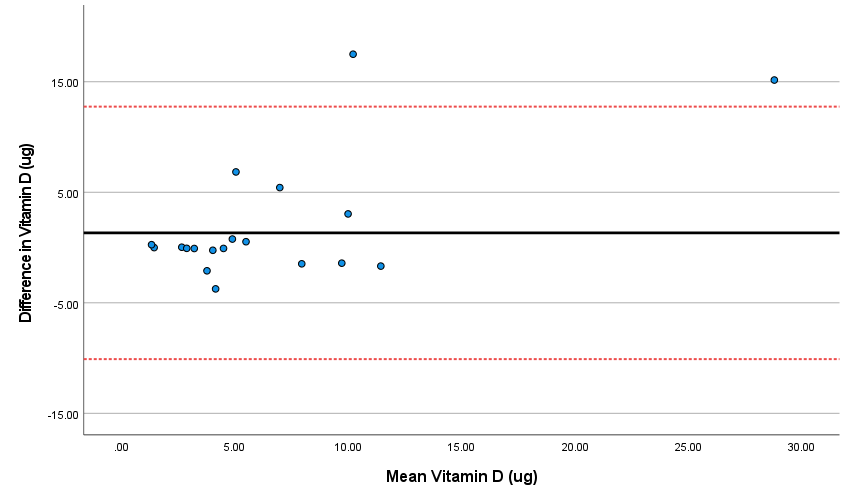


Figure 13. *Difference in vitamin D (ug) reported in the self-administered and interviewer-led recall (Polish sample).*


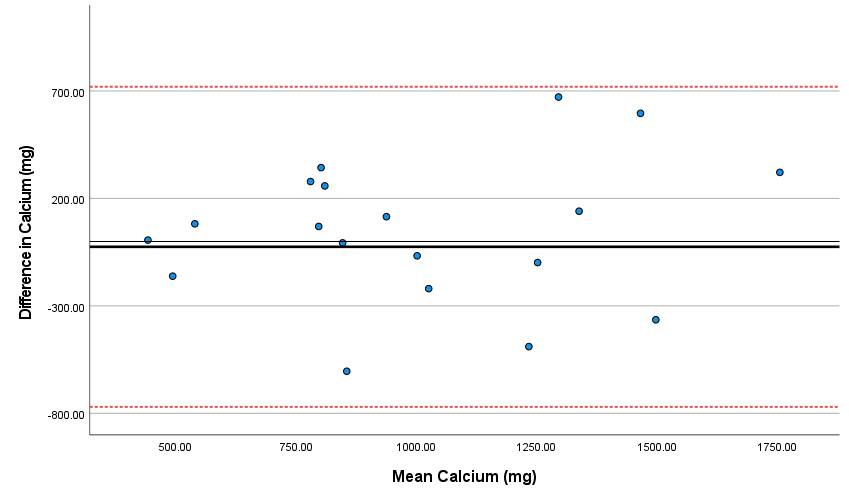


Figure 14. *Difference in calcium (mg) reported in the self-administered and interviewer-led recall (Polish sample).*


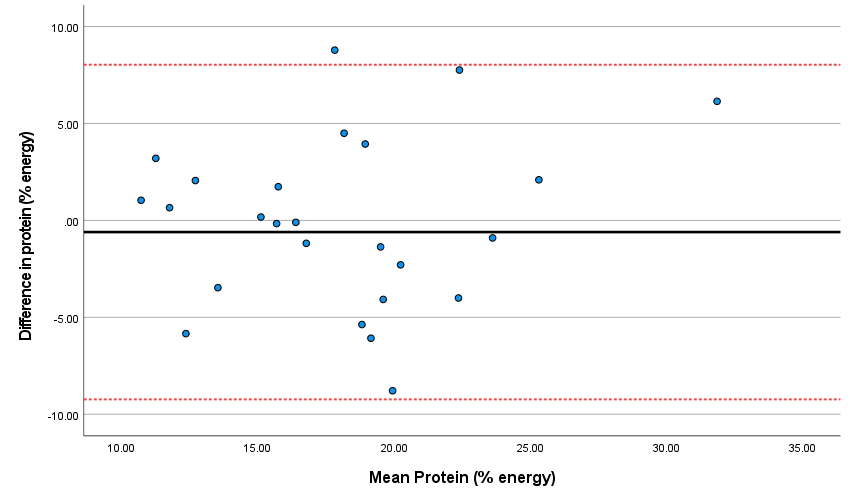


Figure 15. *Difference in protein (% energy) reported in the self-administered and interviewer-led recall (Brazilian sample).*


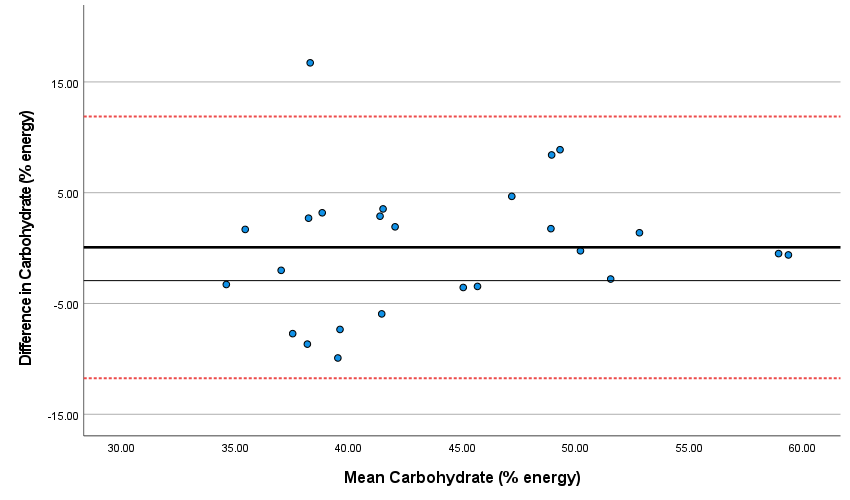


Figure 16. *Difference in carbohydrate (% energy) reported in the self-administered and interviewer-led recall (Brazilian sample).*


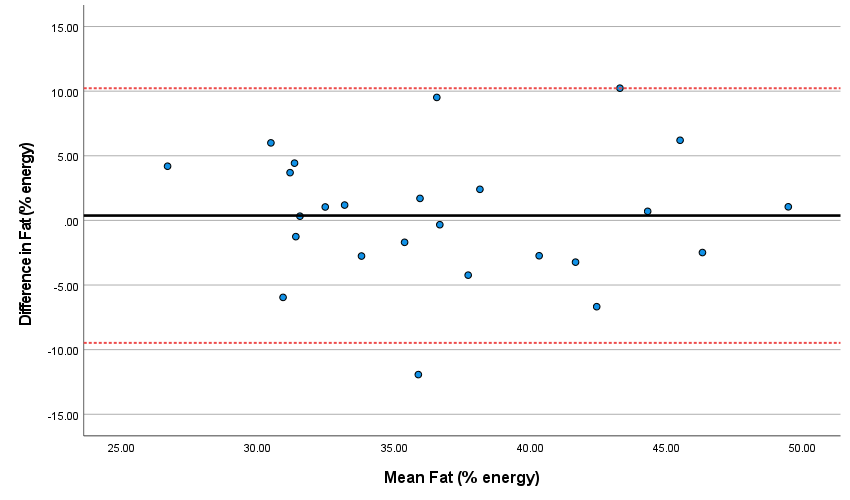


Figure 17. *Difference in fat (% energy) reported in the self-administered and interviewer-led recall (Brazilian sample).*


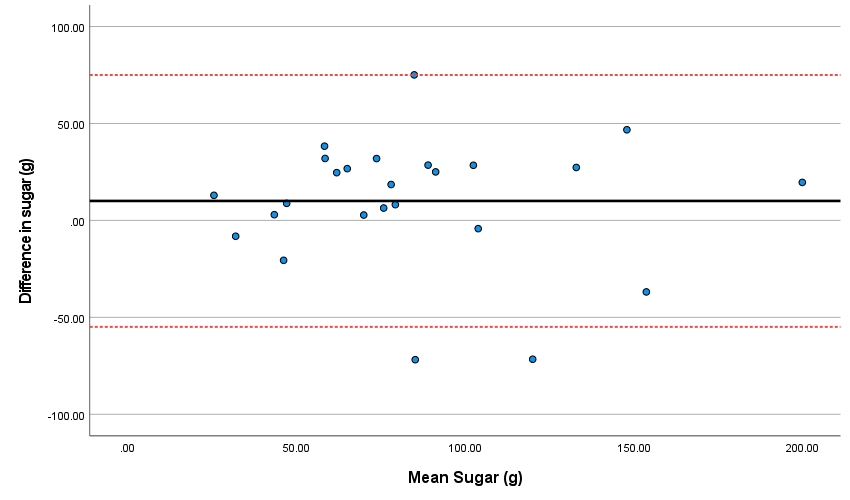


Figure 18. *Difference in sugar (grams) reported in the self-administered and interviewer-led recall (Brazilian sample).*


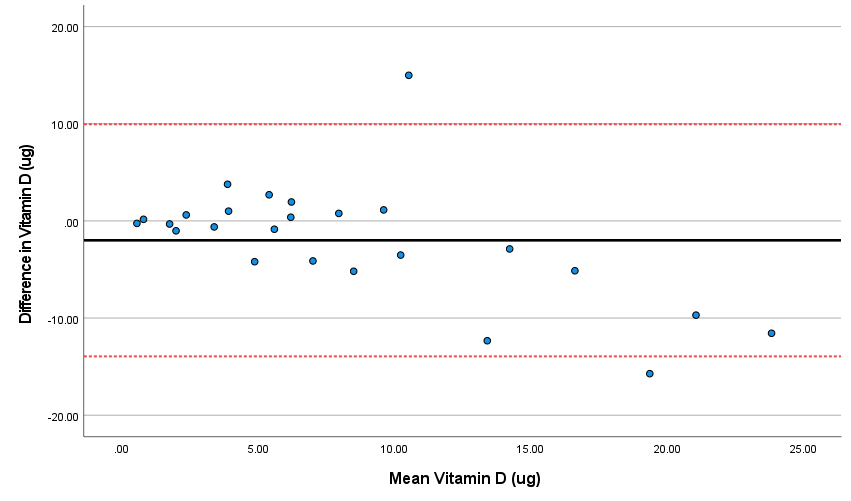


Figure 19. *Difference in vitamin D (ug) reported in the self-administered and interviewer-led recall (Brazilian sample).*


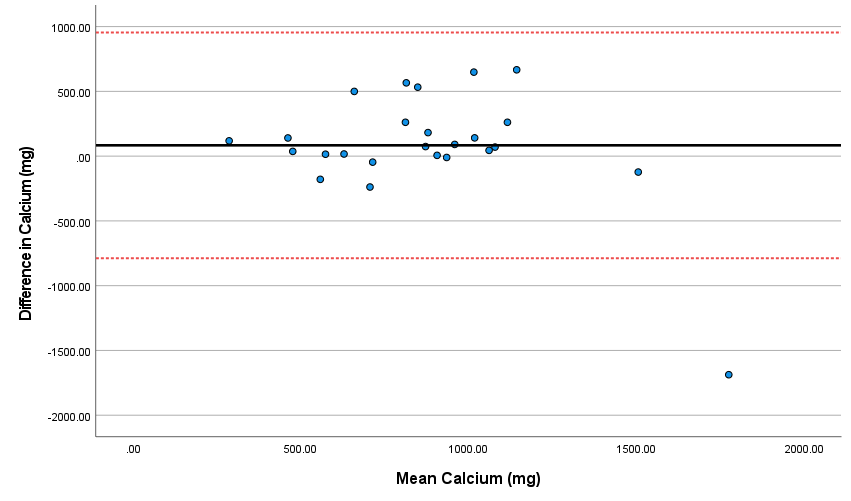


Figure 20. *Difference in calcium (mg) reported in the self-administered and interviewer-led recall (Brazilian sample)*
